# Comprehensive plant disease classification and severity estimation for sustainable farming via automatic segmentation and multi-scale feature fusion

**DOI:** 10.1186/s12870-026-09444-3

**Published:** 2026-07-18

**Authors:** Vinay Gautam, Gaganpreet Kaur, Jyoti Rani, Sathiyaraj Rajendran, G. S. Pradeep Ghantasala

**Affiliations:** 1 Chitkara University Institute of Engineering and Technology, Chitkara University, Punjab, India; 2 Chandigarh University, Punjab, India; 3 Manipal Institute of Technology Bengaluru, Manipal Academy of Higher Education, Manipal, India; 4 Alliance School of Advanced Computing, Alliance University, Bengaluru, Karnataka India

**Keywords:** Apple leaf disease images, Segmentation, Classification, Vision Transformer (ViT), Feature Concatenation, Multi-Scale Feature Fusion (MSFF), Deep learning, CNN

## Abstract

Detecting plant leaf diseases at an early stage is one of the most important requirements for sustainable agriculture, increasing crop productivity, and achieving the global Sustainable Development Goals (SDGs). However, accurately recognizing them in real-world farm fields can still be difficult due to factors such as background complexity, changes in light conditions, and very similar looking classes from a visual standpoint. In order to solve these problems, the authors here present a new Multi-Scale Feature Fusion (MSFF) model that can offer robust and highly accurate performance in identifying plant leaf diseases. Firstly, the brand new hybrid method starts with a U-Net segmentation designed exclusively to separate the diseased parts and thus allow the classification to be more robust. Next, Rank Order Fuzzy (ROF) is implemented to get rid of the background while still maintaining the edges, and the additional data is used for the network to generalize better. The color distribution is then analyzed to determine the variations in color brought about by the infection. In terms of features, EfficientNet is paired with an Attention-based Autoencoder to produce both spatially detailed global features and compact latent representations. The two sets of features are then combined through Canonical Correlation Analysis (CCA) which not only identifies the dependencies between the features but also enhances the discriminative strength. The final fused feature set is fed to module based on YOLO for detection and classification in order to obtain the final result of the plant disease identification system which is both accurate and fast. Among other datasets, the model has been tested on different apple leaf datasets such as FGVC7, AppleLeafSet, PlantVillage Apple, Kaggle Apple Leaves, and ATLDSD, which contain five disease classes. The results of the experiments indicate a classification accuracy of 99.95%, thus the model is superior to several state-of-the-art deep learning and classical machine learning methods. Statistical methods like five-fold cross-validation, paired t-tests (*p* < 0.05), and effect size analysis support the strength and importance of the model. Besides, the Grad-CAM heat maps also reveal that the model is pinpointing the disease areas that are biologically relevant. These findings make it very clear that the MSFF is a reliable, explainable, and scalable tool for precise plant disease recognition even in complex farming environments.

## Introduction

Agricultural product trading is at the verge of major risks due to numerous issues leading to leaf diseases, which in turn are harmful to food security and economic sustainability. Risk assessment for such problems is extremely challenging since they differ depending on the crop, the disease strain, and the farming conditions. Agriculture is the backbone of a nation's economy, not only as the main food provider but also as the largest source of employment for a very large part of the population. It is estimated that around one billion people, or 28% of the global workforce, are engaged in agricultural activities. The biggest producers are countries like the United States, China, and India that have the largest areas of cultivated land. One has to be extremely thorough in all aspects of cultivation including making sure the diseases are prevented in order to be able to produce high crop yields regularly. Pathogens cause such damage to plants that their normal physiological processes are disrupted which leads to reductions both in quantity and quality of the crop. The largest proportion of various plant diseases is due to fungal infections, which stand at over 83%, while viruses and phytoplasmas are each responsible for 9%, and bacterial diseases constitute more than 7% of the cases reported. Traditional methods of diagnosis, even molecular biology methods, can accurately detect pathogens. But, they are costly, require a lot of work, and are often out of reach for many small-scale farmers. Checking the disease by hand, which usually depends on looking at it and the expert's guess, is not feasible on a big scale because human perception is limited, symptoms vary, and it takes time.

So, there is a strong demand for innovative, flexible, and economical technologies to diagnose diseases.

Fruit crops are essential for both health and economic development. They are abundant in minerals such as potassium, folate, and dietary fiber, as well as vital vitamins like C and A. However, they are highly susceptible to diseases that result in significant financial losses annually. For better growing of a wide range of fruit crops, it is important to quickly and precisely recognize leaf diseases. Most fruit crops are grown all over the world in different climates that are important for their nutritional value.

The goal of this study is to find solutions to the problems that these fruit crops face, since they are prone to many kinds of issues. In the past, people dealt with plant problems by looking at their symptoms and patterns by hand. This could hurt the quality and quantity of fruit crops. So, the method has changed to automated leaf disease detection, which marks the beginning of a new era in farming and gardening. In recent years, many studies have looked at leaf images to find problems with plants' leaves, such as diseases. These studies used decision-making systems, predictive modeling, quality control, and soil analysis [1]. Table 1 shows the details of common fruit leaf diseases, including what causes them and what the symptoms are.

**Table 1:** Fruit Plant leaf diseases name with symptoms and causes

Disease Name	Fruit Plants	Symptoms	Cause/Pathogen	Pathogen Type	Mode of Transmission	Optimal Conditions	Impact on Yield	Detection Complexity	Control Measures
Rust Disease	Pear, Plum, Apple	Orange to reddish pustules on leaves	Gymnosporangium spp.	Fungal	Wind-borne spores	Humid, 18–25 °C	Moderate	Medium	Fungicides, pruning infected tissue
Apple Scab	Apple, Pear	Olive-black spots, curled leaves, early defoliation	Venturia inaequalis	Fungal	Wind/rain splash	Cool & moist (12–22 °C)	High (up to 70%)	Medium	Fungicide sprays, resistant cultivars
Bacterial Leaf Blight	Mango, Peach, Plum	Water-soaked lesions turning brown	Xanthomonas spp.	Bacterial	Rain splash, tools	Warm, wet conditions	Moderate	High	Copper-based bactericides
Black Sigatoka	Banana	Black streaks, yellowing, leaf drop	Mycosphaerella fijiensis	Fungal	Wind, rain splash	Warm (27–30 °C), humid	High (up to 50%)	High	Fungicides, defoliation, resistant var
Peach Leaf Curl	Peach, Nectarine	Reddish thickened, curled leaves	Taphrina deformans	Fungal	Airborne spores	Cool & moist (< 16 °C)	Moderate	Low	Dormant sprays, resistant varieties
Cherry Leaf Spot	Cherry, Plum	Purple-brown spots, early leaf drop	Blumeriella jaapii	Fungal	Rain splash	Humid, 18–24 °C	Moderate	Medium	Fungicide applications
Grape Downy Mildew	Grapevine	Yellow oil-like spots, white mold on underside	Plasmopara viticola	Oomycete (fungus-like)	Wind/rain splash	Moist (95% RH), 20–25 °C	High (60% +)	Medium	Copper sprays, canopy management
Fig Rust	Fig	Small reddish-brown spots, defoliation	Cerotelium fici	Fungal	Airborne spores	Warm & humid	Moderate	Low	Fungicides, sanitation

In a realistic scenario, the quality of the plant images was adversely affected by several factors, including fluctuating light conditions and noise, which also hinder the classification performance. In this research, the classification performance was enhanced with the help of various techniques such as better preprocessing, segmentation, multiscale feature extraction and correlation-based feature fusion.

This paper introduces an innovative intelligent end-to-end framework for the analysis of leaf diseases that incorporates pm disease segmentation aware, dual-stream multi-scale feature learning, correlation-driven feature fusion, real-time classification, and severity grading all in a single optimized architecture. Instead of using simple feature concatenation and treating segmentation and severity estimation as separate tasks as done in the case of conventional approaches, the proposed model integrates L*a*b* guided pre-segmentation with U-Net refinement, makes use of complementary global and local representations (EfficientNet + attention-based autoencoder), and utilizes Canonical Correlation Analysis (CCA) for dependency-maximized feature projection. Moreover, a class-wise threshold-based mechanism for severity estimation was added to the system, which makes the diagnosis more clinically relevant. This is a tightly coupled, correlation-aware, and multi-scale system aiming to be a complete and practically deployable tool for precision plant disease diagnostics.

The contributions:

1. First integration of U-Net segmentation with L*a*b* color-guided preprocessing for precise lesion isolation under real-field conditions.
2. Dual-stream feature extraction combining EfficientNet (global context) with an attention-based autoencoder (local discriminative patterns).
3. Canonical Correlation Analysis (CCA) for statistically optimal feature fusion, maximizing inter-set dependencies rather than simple concatenation.
4. YOLO-adapted classification head with integrated class-wise severity estimation (mild/severe/critical) without additional segmentation masks.

The rest of the paper sections are arranged as follows: Sect. " Related work" is focused on describing the most recent articles using which the plant diseases identification has been done. Material and methods under which the study has been conducted are discussed in Sect. " Material and method". Sect. " Experiment results analysis and discussion" is devoted to the Results and Discussion part where data have been analyzed and talked about. Managerial implication and conclusion part, have been discussed in Sect. "Conclusion".

## Related work

Properly identifying healthy and diseased leaves is one of the effective ways for reducing crop loss [2] and ensuring a higher agricultural yield. This part, which concentrates on environmentally friendly farming, reviews the latest deep learning (DL) and artificial intelligence (AI) approaches that are being used to detect and classify plant diseases[3–5]. In order to deal with the class imbalance problem in the recognition of citrus diseases, [6] proposed an EfficientNet-B5 model integrated with SMOTE and data augmentation techniques. The method here proposed not only attained an accuracy level of 99.20% but also markedly outperformed other methods. Considering the general field of the work, apple leaf disease identification using various techniques was the subject of the investigation [7]. The authors' aim was to discuss the reasons for and the impacts of the disease that lowers the quality of apples. [8] gathered a hog plum leaf image dataset for the purpose of identifying plum leaf disease. Comprised of 3782 labeled samples obtained in the wild, this dataset has been used in the effort to treat the leaf disease problem and thus to foster sustainable agriculture. In [9], an advanced disease detection system that can identify diseases in a real-world environment was developed and it achieved an accuracy rate of 95.62%. The approach was tested on both web and mobile platforms so as to thoroughly evaluate the deep learning model. EQID was introduced in [10] utilizing quantum computing to enhance detection accuracy. The proposed model demonstrates exceptional performance, surpassing that of other models. An anticipated model in [11] was a collaboration of two renowned deep learning models, enhanced by improved UNet segmentation. The proposed model outperformed others with an accuracy rate of 99.52%, an IoU of 99.1%, and a recall rate of 99.53%. Hansan S. et al. [12] present an innovative technique that integrates the color segmentation with a modified "Discrete Wavelet Transform (DWT)" algorithm to detect diseased parts of apple leaves. The approach first includes the preprocessing of images and color-based segmentation to isolate the diseased areas accurately. Important features are finally extracted with the modified DWT, and to classify them machine learning approaches were used.

The innovative disease detection model anticipated precision agriculture, reducing the use of pesticides and waste production while enhancing food security. This aligns with the principles of sustainable agriculture and assists farmers in utilizing resources more effectively and efficiently.

Recent advancements in plant leaf disease detection have significantly improved the efficiency and accuracy of intelligent agricultural monitoring systems through the integration of deep learning, hybrid optimization strategies, and attention-based architectures. Developing robust models that can accurately detect diseases in different crop species by keeping the classification efficiency intact even when applied in a real-world environment has become the prime research focus of many researchers. The review papers [13, 14] show that CNNs, transfer learning, hybrid feature extraction, quantum-inspired learning, and ensemble structures are the most common techniques being adopted for precision agriculture applications.

Automated plant disease recognition has taken a new lead with the coming of Deep Learning which is considered as one of the best methods mainly because it can produce discriminative features from leaf images without needing any human intervention [15]. Banjar A. et al. [13] developed an upgraded EfficientNetV2-based model for apple leaf disease classification by employing a specially prepared apple leaf dataset. Their model reached a very high accuracy rate of 99% with a very close F1-score of 98.96%, which proved the great potential of lightweight but powerful CNN architectures in detecting agricultural diseases. The research emphasized EfficientNetV2's ability to A lot lessen computational expenses and at the same time, deliver high classification accuracies. Unfortunately, the method they suggested depended solely on the apple leaf dataset and wasn't tested on different crop environments; So its applicability was limited.

Also, Butt N. et al. [16] came up with an advanced hybrid structure that combined DenseNet-201 AlexNet Moth Flame Optimization (MFO), and Support Vector Machine (SVM) classifiers, to detect diseases in citrus leaves. The model obtained an outstanding 99.6% accuracy and 99.56% F1-score on the Citrus Leaf Database by mixing deep feature fusion with optimization strategies. Still, the setup was computationally intensive and required longer training periods since it required the integration of multiple deep learning components and optimization algorithms. However, Sundaramoorthi et al. [17] focused on the IDLFOA-DCTLFD model, a hybrid structure combining Long Short-Term Memory (LSTM), CNN, and attention mechanisms to identify diseases in tomato leaves. The system reached 98.02% accuracy and an F1-score of 97.88%. They pointed out sequential feature learning and attention-based feature refinement as crucial for disease recognition. Also, the attention mechanisms allowed the model to zero in on the key diseased areas in the leaf images. Even so, the model's computational cost stayed sufficiently high to restrict its use on agricultural devices with limited resources.

Oliullah et al. [18] suggested a new fusion-based design by integrating EfficientNetB7 with ResNet50 for the classification of multiple plant diseases in a dataset of fruits and vegetables. They got 98.96% accuracy and 98.9% F1-score using transfer learning and feature fusion techniques in large agricultural datasets. Their research highlighted the adaptability of these techniques and stronger feature representation in different plant species. Yet, combining several deep architectures increased the inference time and model complexity, which resulted in the least efficient real-time deployment. Shinde et al. [19] investigated and compared traditional machine learning with deep learning methods in analysing CNN, Random Forest (RF), and Simulated Annealing Algorithm (SVM) for disease detection in apple, potato, and strawberry crops. They used Kaggle datasets plus the customized ones and their study showed the highest accuracy of 94.38% with the F1-score of 93.45%. This work sheds light on the results of conventional machine learning and deep learning when it comes to the weakest and strongest models. The models were easier to understand and comparatively simpler but their performances weren't on par with the most advanced deep learning architectures.

Chavan P. et al. [20] created a system that combined Enhanced U-Net, DenseNet-77, and CS-AGRU to simultaneously segment and classify crop pest regions. They were able to gain 99.52% accuracy and 99.35% F1-score with the use of their own pest database. This research highly emphasized the segmenting ability and recall performance of the method. Rahman K.N. et al. [21] came up with a new CNN tailored for use with web and mobile applications integrating real-time features for multiple crops disease detection. The system was tested on the Bangladesh agricultural data set and accomplished 95.62% accuracy and 95.15% F1-score. The researchers targeted ease of use, accessibility, and real-time disease diagnosis for farmers, putting special emphasis on these features. But based on limited training samples and insufficient dataset diversity, the system was of moderate accuracy.

Quantum-inspired deep learning, a new method has also been gaining attention in the smart agriculture domain. Attri I. et al. [14] introduced an Enhanced Quantum-Inspired Deep Learning (EQID) system for the detection of diseases in tomato and potato plants. The system reached an incredibly high level of precision with 99.61% accuracy for detecting diseases in tomatoes and 98.96% for classifying diseases in potatoes. Besides that, the F1-score was at 99.55%. The research work revealed that quantum-inspired feature representations have an influential potential in the feature extraction and classification aspects. Yet, the implemented model have been quite complex and at present, the practical feasibility for real-time agricultural use is excluded.

Table 2 Provided detailed information about a range of the methods.

**Table 2:** Common leaf disease with accuracy

Ref. No	Author(s) & Year	Image Type	Model/Technique Used	Dataset Used	Accuracy (%)	F1-Score (%)	Pros	Cons
[13]	Banjar A. et al. (2025)	Apple Leaf	Enhanced EfficientNetV2 for classification	Custom Apple Dataset	99	98.96	High precision; Efficient model; Works well on apple dataset	Limited to apple leaf dataset; needs generalization
[16]	Butt N. et al. (2025)	Citrus Leaf	DenseNet-201 + AlexNet + MFO + SVM	Citrus Leaf DB	99.6	99.56	Deep feature fusion improves classification; Handles imbalance	Complex model with high training time
[17]	Sundaramoorthi et al. (2025)	Tomato Leaf	IDLFOA-DCTLFD (Hybrid LSTM-CNN-Attention)	Tomato Disease Dataset	98.02	97.88	Robust detection; Good for sequential image analysis	High computational cost; Custom dataset only
[18]	Oliullah et al. (2025)	All Plant Leaves	EfficientNetB7 + ResNet50	Fruits & Veg Dataset	98.96	98.9	Strong performance; Scalable fusion approach	Fusion increases model complexity; slower inference
[19]	Shinde et al. (2025)	Apple, Potato, Strawberry	ML & DL models (CNN, RF, SVM)	Kaggle + Custom Datasets	94.38 (Apple)	93.45	Simple and interpretable; Diverse model comparison	Less accurate than state-of-the-art DL methods
[20]	Chavan P. et al. (2025)	Crop Pest Region	Enhanced U-Net + DenseNet-77 + CS-AGRU	Custom Pest DB	99.52	99.35	Best for segmentation; High recall; Effective on pests	Not tested across plant types; High memory requirements
[21]	Rahman K.N. et al. (2024)	Multiple Crops	Custom CNN + Real-time Web/Mobile Integration	Bangladesh Dataset	95.62	95.15	Practical deployment; Covers multiple crops	Moderate accuracy; limited training data
[14]	Attri I. et al. (2025)	Potato, Tomato	Quantum-Inspired DL (EQID)	Tomato & Potato Dataset	99.61 (T), 98.96 (P)	99.55	Cutting-edge accuracy; Quantum features enhance model depth	High system complexity; Not yet practical for field deployment

Generally, the papers analyzed show that combining hybrid deep learning architectures with transfer learning, attention mechanisms, and optimization-oriented systems has led to drastic improvements in the accuracy of detecting plant diseases. In fact, most the papers presented classification accuracies exceeding 95%, which is a testament to how deep learning can come to the aid of precision agriculture. However, challenges like, the high demand for computing resources, small dataset diversity, weak generalization capability for different types of crops, absence of federated or decentralized learning settings, and very scarce real-world deployment validation still keep coming up as issues that need to be addressed. These factors point towards the need for designing lightweight, scalable and general intelligent systems that could still run smoothly in varied agricultural ecosystems, protect data privacy and support collaborative learning among distributed farms. So, the suggested MSFF is a thoughtful conceptualization of a system which resolves the issues that have hindered the progress of research so far. In fact, the proposed system is a clean labored scalable interpretable, and highly accurate solution for the detection of plant diseases that can efficiently operate in real-field agricultural environments.

## Material and method

### Dataset collection

The dataset used here was taken from the Apple Tree Leaf Disease Segmentation Dataset (ATLDSD) [22] which made up of images taken in real-field environmental conditions. In contrast to the laboratory-controlled datasets, ATLDSD has a lot of background clutter, illumination variability, shadow effects as well as the lesion regions of a small or irregular shape, thus, making the classification and segmentation tasks more complicated and realistic. This variation not only contributes to the ecological validity of the experiment but also makes the task of correctly diagnosing the disease more challenging. The data set consisted of five diseases: Alternaria leaf spot Rust Gray spot, Brown spot, and Healthy leaves. In the beginning, 3282 original images were gathered, then the data set was increased to 12,122 images through data augmentation and normalized. The original data set has separated into 9825, 961 and 1336 images for training, testing, and validation, respectively shown in Table 3.

**Table 3:** Class-wise distribution of images in the dataset showing the total number of samples available for each apple leaf disease category used in training and evaluation

Disease Type	Original Images	Augmented Images	Train	Test	Validation
Alternaria leaf spot	556	2,416	2,000	216	200
Rust	688	2,396	1,906	250	240
Gray spot	790	2,397	1,907	250	240
Brown spot	430	2,457	2,012	245	200
Healthy	818	2,456	2,000	0	456
**Total**	**3,282**	**12,122**	**9,825**	**961**	**1,336**

**Fig. 1:**
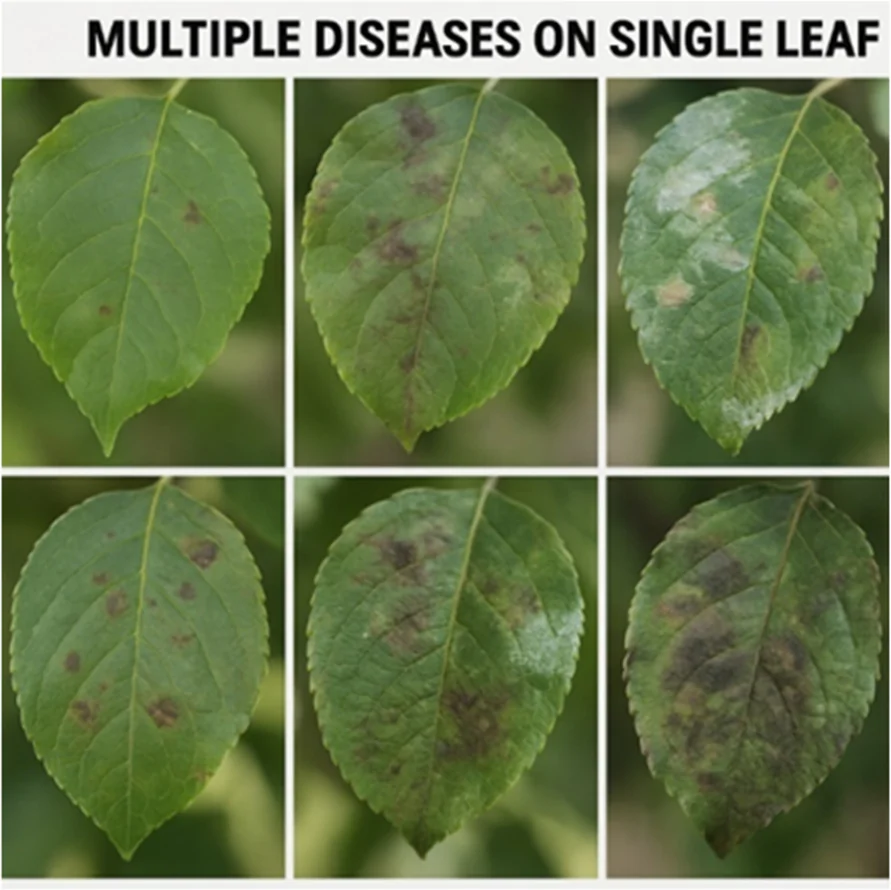
Sample images with Multiple disease

Recognizing and categorizing leaf diseases is quite challenging, in fact, many of them have a very similar appearance. To carry out the task, we decided to resize all the images to three standard dimensions: 256*256, 224*224, and 212*212 pixels. So, by changing the image sizes, we were able to conduct multiple testing of the model performance and enhance its robustness. Besides that, we adopted preprocessing steps like normalization and augmentation aimed at overfitting reduction and better generalization of the model. Figure 1 illustrates some examples of the datasets used in the experiment showing different disease symptoms.

**Fig. 2:**
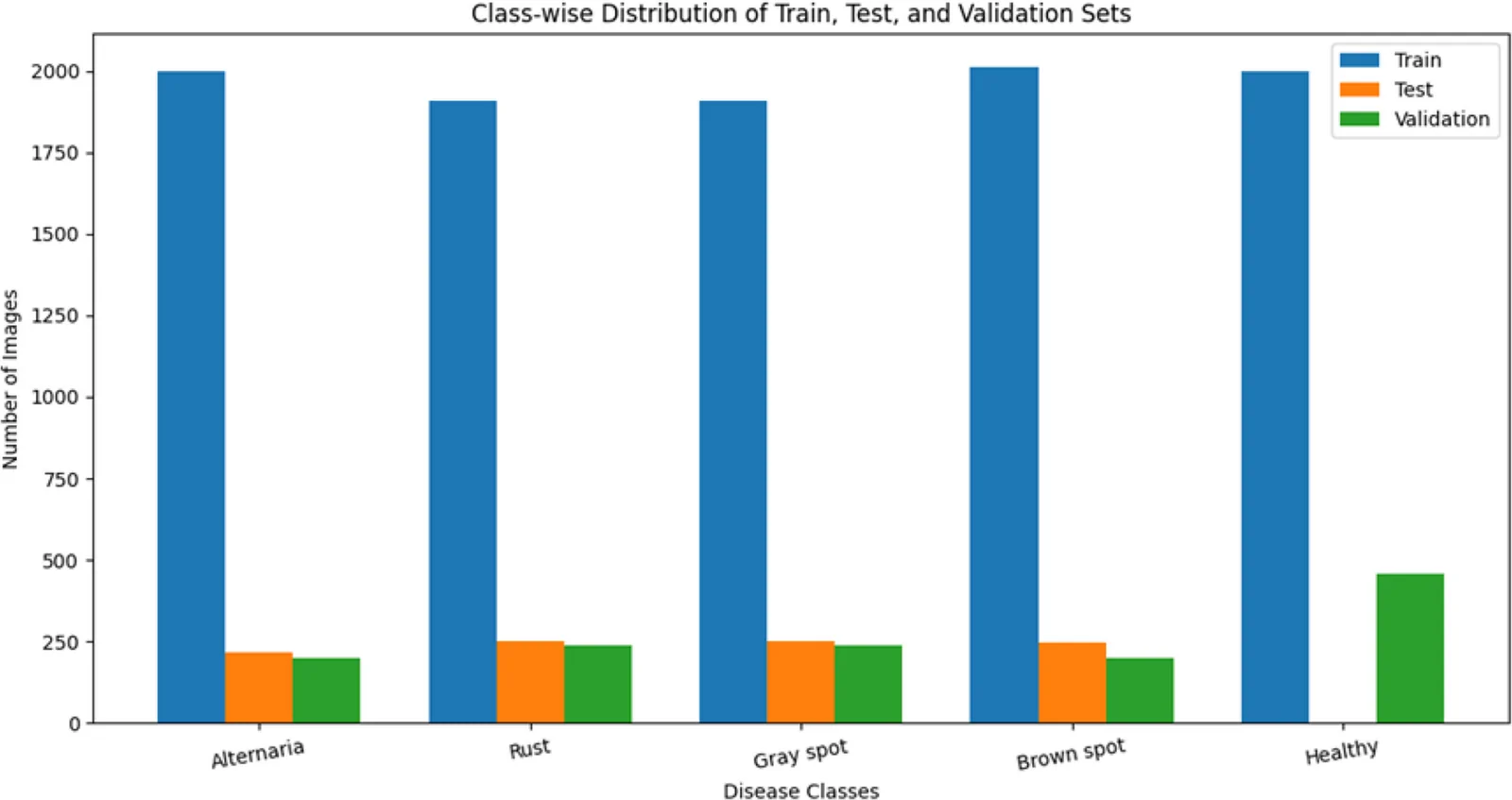
Data balancing

After augmentation, the dataset distribution was carefully balanced so that all classes were equally represented, as clearly seen in Fig. 2. In fact, the training set makes up the largest area. It contains roughly 1,906–2,012 pictures per class, which means that there are enough samples for deep feature learning. Though, testing (216–250 images per class) and validation (200–240 images per class) sets are still relatively small but statistically well-balanced, This way facilitating unbiased evaluation. Such a partitioning scheme is very good for preventing data leakage, removing class bias, and As a result allowing comparative performance assessment across different experimental setups with high confidence.

The original dataset showed a slight class imbalance across the five disease categories, which were Alternaria leaf spot Rust Gray spot, Brown spot, and Healthy leaves. The number of original images per class varied from 430 images for Brown spot to 818 images for Healthy leaves, giving an approximate imbalance ratio (IR) of 1.90. Even though the imbalance wasn't very serious, it's a common thing that deep learning models during training get biased towards majority classes, and this might cause negative effect of minority-class recognition as well as model generalization overall. That means, an extensive set of data augmentation methods was carried out to increase the representation of the different classes as well as to make the dataset more uniform. After augmentation, the class distribution had become very balanced with each class containing roughly between 2,396 and 2,457 images. More in detail, Alternaria leaf spot had 2,416 images, Rust 2,396 images, Gray spot 2,397 images, Brown spot 2,457 images, and Healthy leaves 2,456 images. This balanced augmentation approach lessens class dominance and strengthens the feature learning ability. Also, to enhance the learning of minority classes and help the model differentiate between very similar-looking disease patterns, one may choose to integrate the Synthetic Minority Oversampling Technique (SMOTE) in the deep feature space following CNN-based feature embedding extraction. Instead of creating synthetic examples in the pixel space, this version of SMOTE works with the latent feature vectors derived from the CNN layers. In this manner, embeddings of minority classes like Rust (1,906 training samples) and Gray spot (1,907 training samples) are synthetically increased by interpolating between the feature representations of nearest neighbors within the same class. This method results in a more refined decision boundary and lessens the bias of the classifier to more dominant classes e.g. Brown spot (2,012 training samples) and Healthy leaves (2,000 training samples). Post augmentation and balanced dataset partitioning, the dataset was divided into training, testing, and validation subsets as follows:

- Training set: 9,825 images
- Testing set: 961 images
- Validation set: 1,336 images

The training dataset remains highly balanced across all classes, ranging from 1,906 to 2,012 samples per class, enabling stable and unbiased model learning. Similarly, the testing and validation sets preserve proportional class representation, thereby ensuring fair and reliable evaluation. The testing samples vary between 216 and 250 images per class, while validation samples range between 200 and 456 images.

The integration of augmentation, balanced dataset splitting, and optional feature-level SMOTE collectively provides a statistically reliable framework for mitigating class imbalance in mango leaf disease classification. This strategy offers several important advantages, including:

- Improved minority-class recall
- Reduced classifier bias toward dominant classes
- Enhanced macro-averaged F1-score
- Better feature generalization for subtle disease variations
- Increased robustness and stability during model training

Consequently, the proposed balancing strategy significantly improves the effectiveness and reliability of deep learning-based mango leaf disease detection and classification systems.

### Proposed methodology

**Fig. 3:**
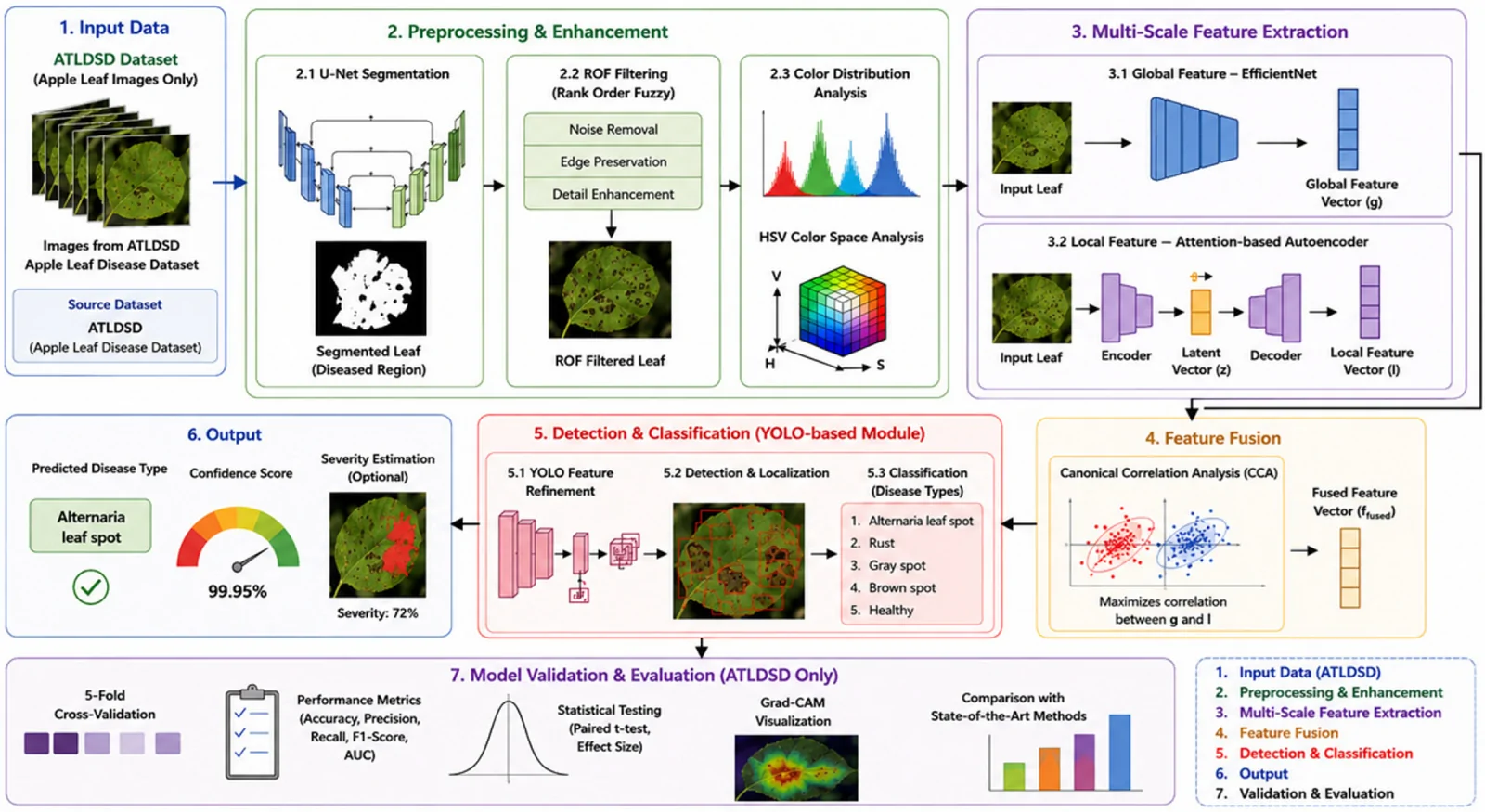
Improved architecture of the proposed Multi-Scale Feature Fusion (MSFF) framework, where global features are extracted using EfficientNet and local discriminative features are derived through an Attention-based Autoencoder. The complementary representations are fused via Canonical Correlation Analysis (CCA) and forwarded to a YOLO-based classification module for accurate apple leaf disease detection

Leaf disease was detected by multi-scaled feature fusion methods. These methods go through steps such as segmentation and multi-scale feature extraction, and finally, feature fusion. This method has great potential in classification but still, it cannot make predictions in real-time and detect diseases at the early stages very accurately. The overall structure and process of the proposed system are shown in Figure 3.

#### Data preprocessing

In this research, the work focused on the investigation of the ATLDSD [23] apple plant leaf dataset for the purpose of meeting the goal. The leaf pictures of the dataset were preprocessed for the first time to get rid of the blurry and poor-quality photos. Then, the pictures were converted to different resolutions to be able to identify the qualities better as per the experiment specifications. These steps not only maintain the uniformity and compatibility of the dataset with a deep learning model but also help to improve the classification performance. The explanation of the preprocessing operation is shown in various sub-sections.

##### Noise filtering

Impulsive noise in images is a real tough problem which here is tackled by the rank order fuzzy filter. This filter makes use of advanced fuzzy logic and rank ordering criteria to sort the pixel intensities in a certain region. Then the ROF filter decides on pixel values based on their membership in intensity groups, thus enabling a more efficient and flexible reconstruction process [24–26]. The ROF filter corrects erroneous pixel values based on their rank values, as illustrated in Eq. (1).

1 $$\begin{aligned} y_{x,y} =\left\{\begin{array}{cc}\sigma\times B_{x,y}+ [\![1-y_{y}]\!] \times R_{x,y} & where\ \sigma>0.7\\ \sigma\times N_{x} & where\ \sigma \leq 0.7 \end{array}\right\} \end{aligned}$$

**Fig. 4:**
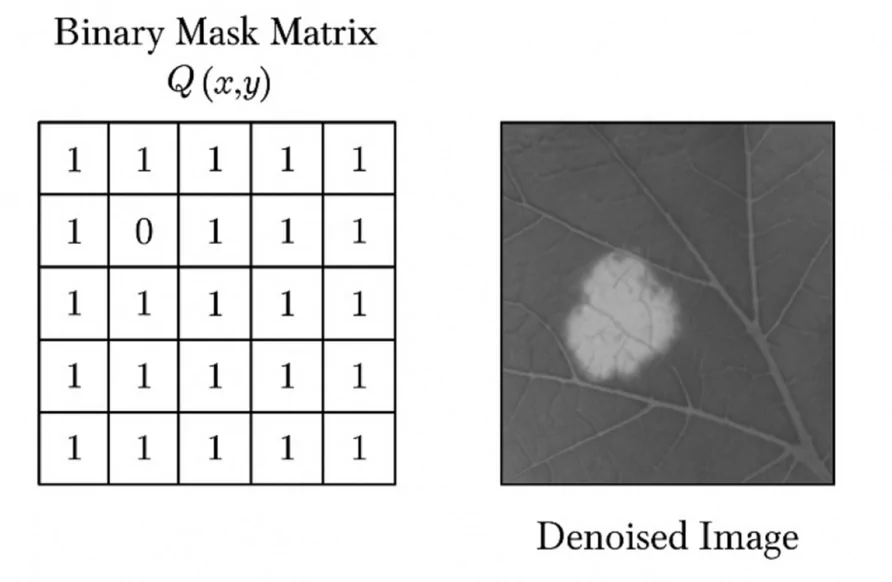
Set the corrupted pixel intensity and de-noised the image

Design a 2D matrix with ${Q}_{xy}$ index element values of either 0 or 1. The following procedure determines if an index in an index matrix ${Q}_{xy}$ is 0 or 1: Make a $\left(2M{ }_{t}\times 1\right)\left(2M{ }_{t} \times 1\right)$ neighborhood area with ${Q}_{x,y}$ at its center for each pixel ${Q}_{xy}$ at position $(\mathrm{x}, \mathrm{y}$) in the picture. The corrupted pixel are in between 0 or 255, but the condition are corrupted pixel are set the binary value as ${Q}_{xy}$ = 0 otherwise set as 1. Figure 4 shows the de noised image after implementing using ROF.

The following equations(Eq. (2) and (3)) represent the binary corruption detection process used in Rank Order Fuzzy (ROF) filtering for identifying noisy and clean pixels.

**Equation (** 2 **): Corrupted pixel detection**

2 $$Q\left(x,y\right)= 0 if I\left(x,y\right)= 0 or I\left(x,y\right)= 255$$

This equation identifies corrupted pixels affected by salt-and-pepper noise. Pixels having intensity values equal to 0 or 255 are treated as noisy pixels.

**Equation (** 3 **): Uncorrupted pixel detection**

3 $$Q\left(x,y\right)= 1 if 0 < I\left(x,y\right)< 255, \forall \left(x,y\right)\in \Omega$$

This equation identifies clean or uncorrupted pixels. All pixels whose intensity values lie between 0 and 255 are considered noise-free.

where:

- Ω is the image domain,
- I(x,y) is the pixel intensity at coordinate (x,y),
- Q(x,y) indicates whether a pixel is corrupted (0) or clean (1),
- A corrupted pixel is defined as having an intensity of 0 or 255 (salt-and-pepper noise),
- All other pixels are treated as uncorrupted.

**Fig. 5:**
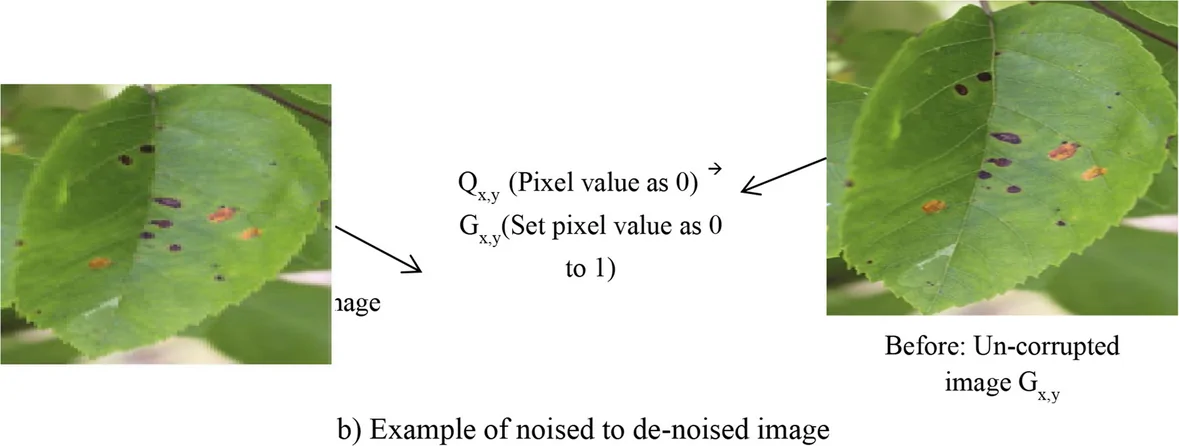
Set noised to de-noised the image **a**) Set the corrupted pixel intensity and de-noised the image **b**) Example of noised to de-noised image

Additionally, for denoising, a local neighborhood 𝒩ₓᵧ centered at pixel (x,y) with size (2Mₜ + 1) × (2Mₜ + 1) is considered and depicted in Eq. (4) and Fig. 5:

4 $$\begin{aligned} N_{xy} &=\left(\mathrm i,\mathrm j\right)\in\Omega |\mathrm x-\;{\mathrm M}_{\mathrm t}\;\;\leq\mathrm i\leq\times\\ &\quad +{\mathrm M}_{\mathrm t},\mathrm y-{\mathrm M}_{\mathrm t} \leq\mathrm j\leq\mathrm y+{\mathrm M}_{\mathrm t} \end{aligned}$$

If $S\left(\frac{\beta}{2}+1\right)\leq\;Q_{x,y}\;\leq S\left(n-\;\left(\frac{\beta}{2}\right)\right)$ When α is an integer and $0 \le\upbeta \le \mathrm{n},$ is set to 0 and ${Q}_{x,y}$ is an uncorrupted pixel. Determine the predicted percentage of impulse noise by applying the technique in Eq. 5 [27]:

5 $$\upmu =\frac{1}{UV}{\sum}_{x=1}^{U}{\sum}_{y=1}^{V}{G}_{xy}\times 100\%$$

Otherwise, the value of ${Q}_{x,y}$ is set to 1 and the pixel is changed.

##### Image resizing

**Fig. 6:**
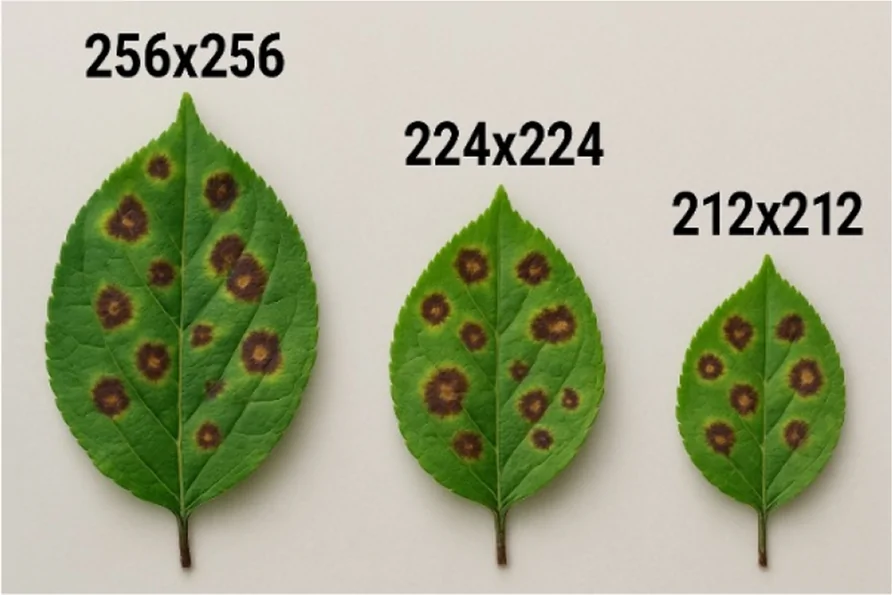
Resizing the apple image data

To preserve the dataset, it is essential to implement scaling, normalization, and augmentation techniques, as certain images in the ATLDSD collection are fuzzy and damaged. All leaf images were resized to 224 × 224 × 3 pixels to ensure uniformity and compatibility with the model. Reducing scaling is necessary to standardize and ensure consistency in input dimensions, as well as to decrease the computational complexity of the framework [28]. Figure 6 illustrates an example of scaled apple image data, reduced from 512 × 512 × 3 to dimensions of 256 × 256, 224 × 224, and 212 × 212 pixels.

##### Data normalization and image augmentation

Normalization is performed using the Min–Max scaling method to enhance the quality and uniformity of the dataset. This technique modifies the pixel intensity values of the image data to a range of 0 to 1, thereby enabling binary encoding. The normalized value *T*β for each pixel is determined by Eq. 6 [29]:

6 $${T}_{\beta }=\frac{{R}_{\beta }-\mathrm{min}\left({R}_{\beta }\right)}{\mathrm{max}\left({R}_{\beta }\right)-\mathrm{min}\left({R}_{\beta }\right)}$$

$where\, {R}_{\upbeta }\,\mathrm{r}\mathrm{e}\mathrm{p}\mathrm{r}\mathrm{e}\mathrm{s}\mathrm{e}\mathrm{n}\mathrm{t}\mathrm{s}\, \mathrm{t}\mathrm{h}\mathrm{e}\, \mathrm{o}\mathrm{r}\mathrm{i}\mathrm{g}\mathrm{i}\mathrm{n}\mathrm{a}\mathrm{l}\, \mathrm{p}\mathrm{i}\mathrm{x}\mathrm{e}\mathrm{l}\, \mathrm{v}\mathrm{a}\mathrm{l}\mathrm{u}\mathrm{e}$ and $\left\{\mathrm{m}\mathrm{i}{\mathrm{n}\left(R\right\}}_{\upbeta }\right) \mathrm{a}\mathrm{n}\mathrm{d} \left\{\mathrm{m}\mathrm{a}{\mathrm{x}\left(R\right\}}_{\upbeta }\right)$ depict minimun and maximum pixel value of an image.

The model's learning experience is made more robust and varied by employing a variety of data augmentation approaches after normalization. The general quality of the pictures is also improved by augmentation. The dataset undergoes many adjustments, including translation, scaling, and luminance alteration after initially being received from the Crop Leaf Dataset.

### Segmentation and patch creation

To increase the efficiency of CNN model use object segmentation technique. The eradicate the object from image from image done by using segmentation technique. So, segmentation is process for elimination the disease object from image. It is necessary since the feature extraction procedure may become biased due to the added background data. Our proposed segmentation process consists of two steps: a) segmenting ill regions and b) generating an average color marker. Furthermore, Segmenting ill region and create color marker are discussed in next section.

In order to enhance the efficiency and precision of the classification process, we incorporate an object segmentation technique utilizing the U-Net architecture, which is a well-recognized convolutional neural network specifically designed for biomedical image segmentation. Segmentation is a vital step in the considered system because it helps to accurately distinguish the disease spots from different parts of the background. When there is a shadow or soil or even some healthy parts of the leaf, it can become confusing for feature extraction and classification as these elements act as noise and bias. Concentrating only on the infected areas through segmentation, the model is directed to the correct parts of the image that have diagnostic significance, thus discriminative learning is enhanced, and the overall classification becomes more stable. In fact, it is quite necessary when the symptoms of the disease are breaking out in a very slight way or very similar to the healthy tissue. The segmentation pipeline that is suggested in this paper is the one that is built upon a U-net and includes two main stages:

1. Segmentation of diseased regions:
  U-Net carries out pixel-level semantic segmentation that is extremely detailed and accurate when it comes to locating the infected parts in the leaf tissue. Its encoderdecoder structure, coupled with the use of skip connections, manages to retain the level of detail through maintaining the spatial resolution while extracting the lowest-level features of texture and highest-level of contextual information. The construction of the network is such that it is able to pinpoint the boundaries accurately and at the same time, it can characterize the disease patterns very well that possibly have a high degree of complexity.
2. Extraction of Average Color Marker:
  After performing segmentation, the color features of the separated infected area are investigated to determine an average color marker. This quantitative descriptor records the color changes related to the infection and acts as an extra discriminative feature at the time of classification. Using this color-based marker thus improves the model's capacity to differentiate between visually similar disease classes.

All in all, the combination of U-Net-based segmentation with feature-level analysis reinforces the trustworthiness and clarity of the disease classification framework. This U-Net-based segmentation framework not only gets rid of image data that isn't useful, but it also makes sure that the next steps of feature extraction and classification work on clean, disease-focused image data. The next section will go into more detail about the U-Net model's full architecture and parameters, as well as how it is trained and tested.

#### Multi object discrimination with UNet

It is quite challenging to distinguish between various types of diseases using only a single image of an apple leaf, as the symptoms and visual characteristics frequently overlap. To solve the problem of having more than one disease on a single leaf, the method uses a UNet segmentation framework that finds disease areas and puts them into different disease groups at the same time. This challenge is about segmenting multiple objects, which means putting diseases into different groups, as shown below:

- Class 0: Background (R3)
- Class 1: Leaf Tissue That Is Healthy (R1)
- Class 2: Region That Is Rust-Infected
- Class 3: Region That Is Scab-Infected
- Class 4: A place with both rust and scab (R2)

Below is a full description of the algorithm:

**Figure:**
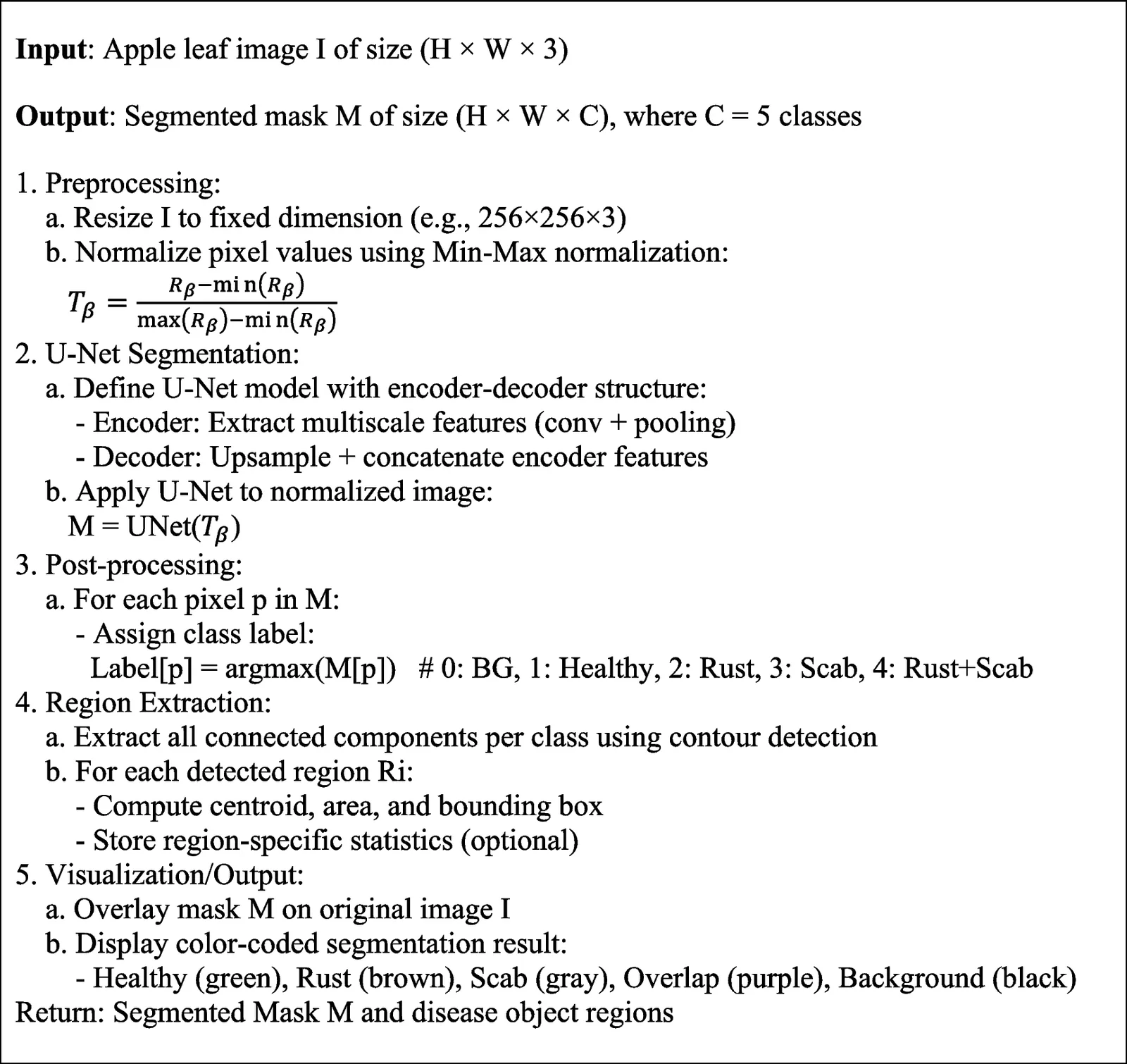
**Algorithm**: multi-disease segmentation using U-Net

The multi-disease segmentation algorithm is designed to detect and sort the various diseases on apple leaves by a U-Net architecture. It first takes a leaf image as input, preprocesses it by cropping it, resizing it to a fixed size (say, 256,256), and then normalizing its pixel values with min–max normalization—independently scaling each color channel between 0 and 1. The resulting normalized image is then fed to a U-Net architecture, a convolutional neural network that has an encoder-decoder structure. The encoder extracts features at multiple resolutions through a series of convolutions and pooling, while the decoder upsamples the features and combines them with the encoder outputs to produce a segmentation mask. In the resultant mask, each pixel is assigned a label according to the most probable class (taken by argmax), thus separating background, healthy tissue, rust, scab, or overlapping infections.

Finally, in the post-processing step, the disease-specific regions are isolated by extracting contours of each connected component. Thus, for each region, the spatial features like the centroid, area, and bounding boxes are derived that can be used for further disease investigation. Finally, for visualizing the results, a color-coded segmented mask is overlaid on the original image, where each class is represented by a certain color making it very easy to identify the disease pattern on the leaf.

#### Object segmentation from leaf

In the multi-disease segmentation framework for apple leaf images, the dataset first contains RGB images, which after that are enhanced by converting them to the L*a*b* color space. This color space that is perceptually uniform is very effective in distinguishing the details of disease symptoms, as it separates the image into three separate channels:

- *Il*: Lightness
- *Ia*: Green–red spectrum
- *Ib*: Blue–yellow spectrum

In order to help segmentation and to improve the ability of U-Net to extract features, three polygonal areas are deliberately picked from the typical leaf sample image. These areas are healthy spots on the leaf and serve as the template or standard. The mean color values of all three L*a*b* channels from these healthy spots are taken as color markers. Using the Nearest Neighbor Classification method, every pixel of the L*a*b* image is matched with this reference marker. According to the closest Euclidean distance in color space, pixels are divided into:

- *R*1: Diseased Region — significantly different from ϕ
- *R*2: Healthy Region — closely matches ϕ
- *R*3: Background Region — non-leaf or neutral area

This initial classification step facilitates the U-Net segmentation stage in two major ways:

- U-Net training is improved with less false positives by providing prior localization clues.
- It makes a more precise post-processing possible to check U-Net results using L*a*b*-based color clustering.

**Fig. 7:**
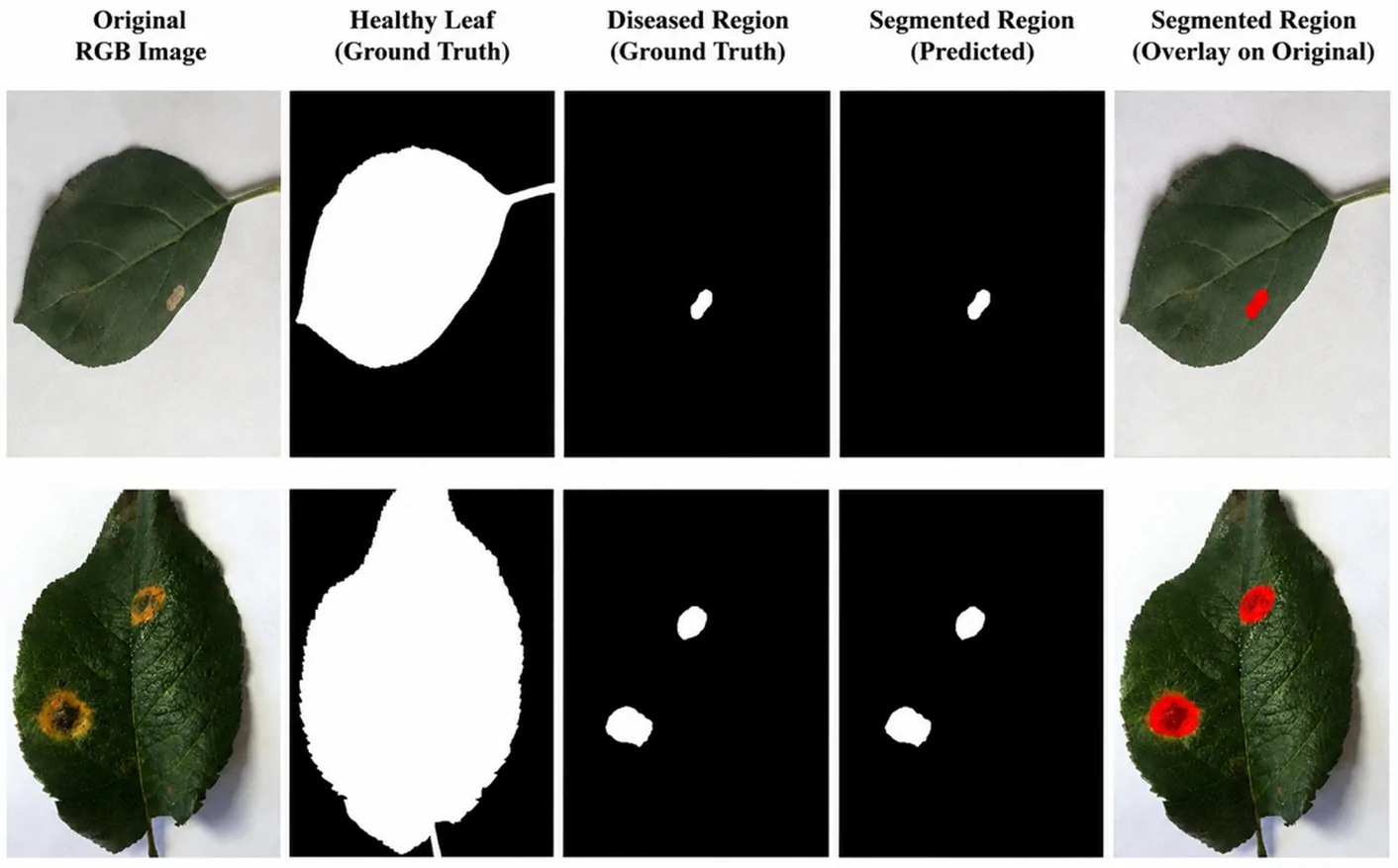
Healthy, diseased, and segmented region of apple leaf image

After this color-based region separation, the U-Net model, which is trained on either normalized RGB or L*a*b* inputs, performs pixel-wise segmentation of the disease categories. Following segmentation, connected component analysis is applied to identify disease spots, calculate features such as area and centroid, and depict the results with a color-coded overlay as shown in Fig. 7. This combined method ensures the accuracy of deep learning techniques and the interpretability of color-based diagnostics for apple leaf disease effectively.

- R1​: Diseased Region
- R2: Healthy Region
- R3​: Background Region — non-leaf or neutral area

This pre-classification step supports U-Net segmentation in two ways.

These three areas correspond to the leaf image's healthy and diseased parts, respectively. The background of each part is shown through a different color channel. The preprocessing stage starts with the filtering of leaf images to remove noise and unnecessary background artifacts, thus making the images clearer for the next processing steps. Then, the disease-affected areas are isolated by means of a region-growing method with self-initialized seed point to accurately delineate the disease spots. In order to evaluate the segmentation quality, several well-known performance metrics (such as IoU, Dice Coefficient, Precision, and Recall) are used, and the results show a great level of agreement.

### Feature extraction

After segmentation, local features (texture, boundary edge, and shape of the lesion) as well as global features (color distributions and the extent of lesions on the leaf) are extracted. Features like color and texture are derived from the lesion areas to provide a richer set of features [30]. The features extracted are then used to guide the classification models.

**Fig. 8:**
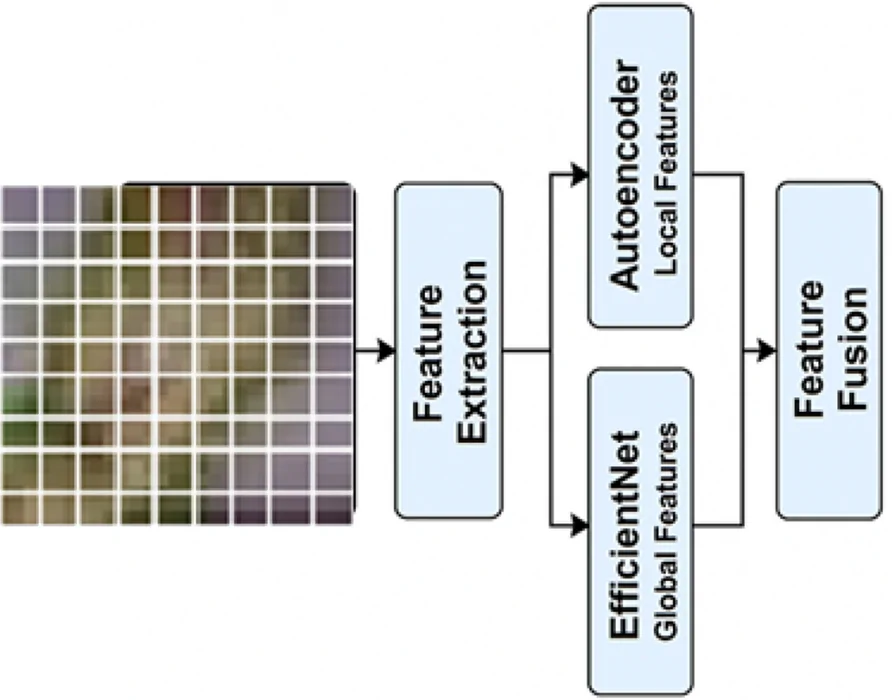
Flow of patch segmentation process

**Fig. 9:**
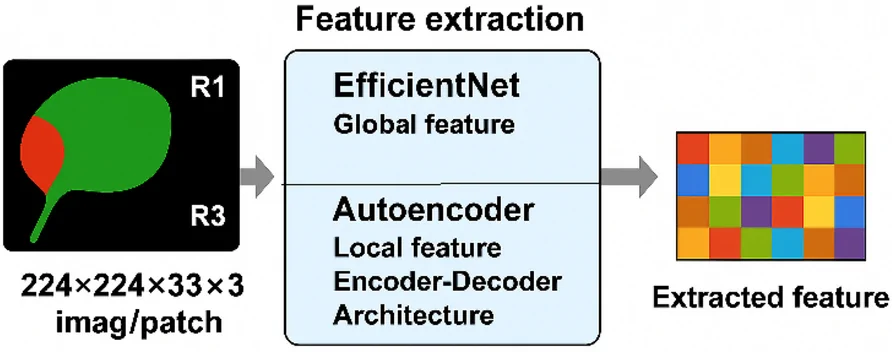
Detail description of feature extraction

To make classification more robust, a combined patch-based and full-image method hybrid strategy has been used. Patches of leaf images coming from the segmentations are then used to capture local distinguishing features. These patches are classified separately and the classification results are combined to predict the global class label of the whole leaf EfficientNet, leveraging its compound scaling technique, is the main backbone used for feature extraction because of its ability to provide a good trade-off between accuracy and computational efficiency at different image resolutions. An Autoencoder-based model is also used alongside to obtain a compressed latent representation of the segmented and preprocessed images. The encoder captures both local and global context information in a compressed form, and the decoder reconstructs image features which helps in unsupervised feature enhancement and anomaly localization. The latent space vectors coming from the Autoencoder are then concatenated with the EfficientNet features to improve the classification performance and the ability to generalize especially in the case of small or imbalanced datasets. The step-wise details of features extraction have been shown in Figs. 8 and 9.

The input leaf disease images are divided into patches that overlap by $\mathrm{p}\times p$. The *16× 16* pixel size patch consists of 1024 pixels; a patch is called as 1 (vertebra or foreground patch). In this work, they used a certain pixel stride for the sliding window to construct overlapping patches for the training dataset. Figure 9 has shown the normal working process of feature extraction.

#### Local feature extraction using attention-based autoencoder

Local feature extraction focuses on capturing fine-grained patterns from small image patches (e.g., 16 × 16 pixels), such as disease-specific spots, textures, or lesions. Each patch is passed through an attention-based autoencoder architecture, which includes an encoder, attention mechanism, and latent representation generator.

**Fig. 10:**
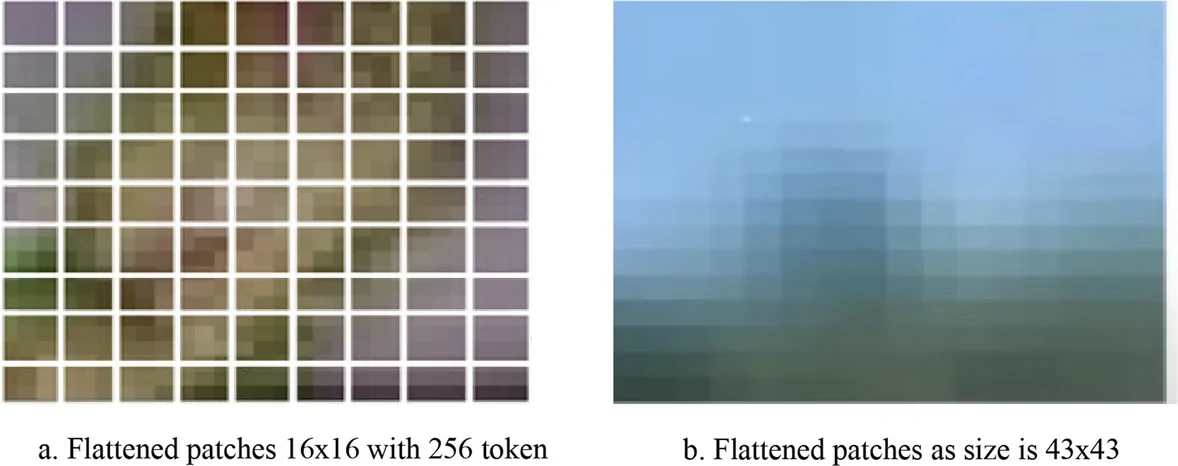
Sample of patches, which have randomly selected by image

The attention-based autoencoder is designed to extract localized features from small image patches (e.g., 16 × 16 pixels) focusing on disease-specific patterns such as lesions and textures. Each input patch is $X_i\in\mathbb{R}^{16\times16}$ first processed by an encoder to produce a hidden representation $h_i=Encoder\;\left(x_i\right)$. To enhance feature discrimination, an attention mechanism is applied, where attention weights are computed as $\alpha_{\mathrm i}=softmax(W_{\mathrm a}\;h_{\mathrm i}+b_{\mathrm a})$ being learnable parameters. These attention weights emphasize the most informative components of the hidden representation. The final local feature vector for patch iii is obtained by applying the attention weights to the hidden representation, resulting in ${\mathrm z}_{\mathrm i}=\alpha_{\mathrm i}\cdot h_{\mathrm i}$ serves as a compact, attention-enhanced descriptor of the patch. The equations were depicted from Eq. 7, 8, 9 and 10 and Fig. 10.

$$Let\,each\, input\, patch\, be:$$

7 $$x_{\mathrm i}\in\mathbb{R}^{16\times16}$$

$$The\, encoder\, generates\, hidden\, representations:$$

8 $$h_{\mathrm{i}}=Encoder\left(x_{\mathrm{i}}\right)$$

$$\begin{aligned}Attention\,mechanism\,is&\,applied\,to\,enhance\\&\,feature\,discrimination:\end{aligned}$$

9 $${\upalpha}_{\mathrm{i}}= {softmax}\ (W_{\mathrm{a}}h_{\mathrm{i}}+b_{\mathrm{a}})$$

$$The\, attention-weighted\, latent\, vector\, is:$$

10 $$\mathrm{z}_{\mathrm{i}}\;=\;\alpha_{\mathrm{i}}\;\cdot\;h_{\mathrm{i}}$$

$$Where\;z_i\in\mathbb{R}^{\mathrm{dl}}\;is\;the\;local\;feature\;vector\;for\;patch\;i.$$

#### Global feature extraction using efficientnet

**Fig. 11:**
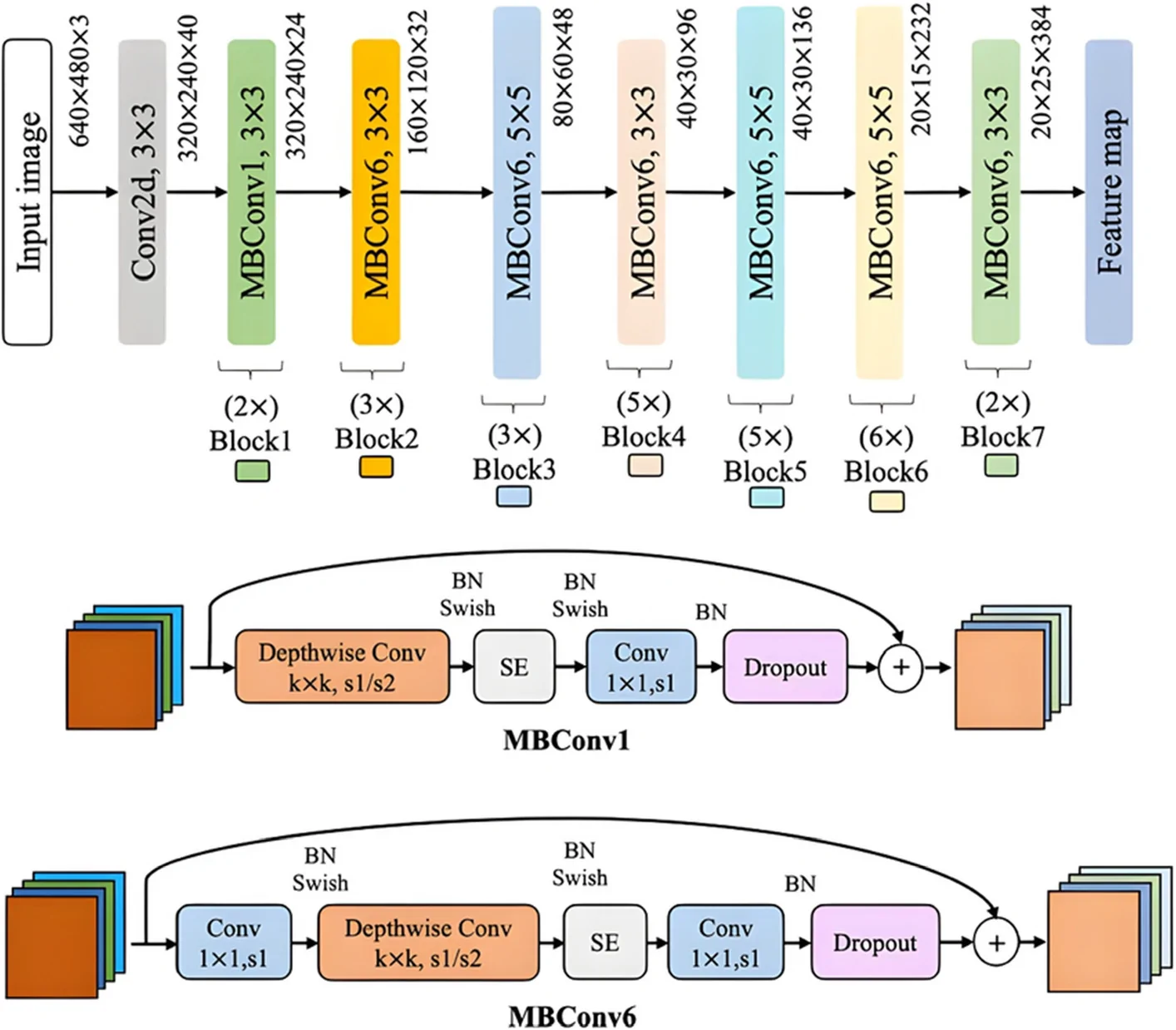
Layered description of EfficientNet

The EfficientNet-B6 model, which is a convolutional neural network (CNN) architecture, is used for plant leaf disease classification due to its outstanding accuracy and computational efficiency as shown in Figure 11. EfficientNet-B6 utilizes a carefully balanced scaling method that simultaneously changes the network's depth, width, and resolution, thus enabling it to capture both fine-grained textures and global context effectively. At first, the input image is resized to the EfficientNet-B6 required resolution (528528 pixels). It then continues through a series of Mobile Inverted Bottleneck After the convolutional backbone, a global average pooling layer is used to reduce the feature maps to a fixed-length vector. This is followed by a dense (fully connected) layer, a Dropout layer to prevent overfitting, and a final SoftMax activation layer that gives class probabilities for disease classification

This design eliminates the need for patch-wise tokenization and positional embeddings since spatial relationships are naturally learned via convolutional operations. An example image fed into EfficientNet-B6 is shown in Fig. 8, which explains how the feature maps evolve from the input to the output at different depths of the network. Global feature extraction is performed using the EfficientNet model that is capable of taking into account the overall structure and contextual information of the entire leaf image.. Suppose the input image is (e.g. after resizing to 224*224*3); the model outputs a global feature vector *F*g = EfficientNet(*I*) ∈ ℝᵈᵍ. This vector encodes the holistic characteristics of the leaf, preserving both contextual and structural information. Following this, global average pooling and a fully connected (dense) layer are applied to obtain a refined global representation g′ = Wg ⋅ *F*g + bg, where *Wg* ​ and *bg* are learnable parameters.

### Multi-scaled fusion

During the training phase, each patch was used as input. It was either stored as a single image file or a NumPy array. After the patches were extracted, the learning model used a Multi-Scale Feature Fusion (MSFF) strategy that included a Canonical Correlation Analysis (CCA)-based fusion mechanism. This method made it possible to combine feature maps from different scales or model branches. We used CCA to put the feature vectors from the EfficientNet and autoencoder pathways into a shared subspace where they are most correlated. Primarily, the collective representation ${F}_{c}=\left[\left(1/N\right){\Sigma}_{i=1}^{N}{z}_{i}\parallel {F}_{g}{\prime}\right]$ is created by putting together the averaged local features ${z}_{i}$ ∈ ℝⁿˣᵈˡ with global features *F*g′ ∈ ℝⁿˣᵈᵍ. Then, CCA learns the projection matrices W_l_ and Wg​ to compute u = Wₗᵀ ${z}_{i}$, v = Wgᵀ *F*g′ so that the correlation ρ = corr(u, v) is as high as possible.

Resultant vector *F*fused = [u, v] ∈ ℝᵈᶜ combine both global and local feature with in a shared space represented with *d*ᶜ that enhance classification accuracy.

**Figure:**
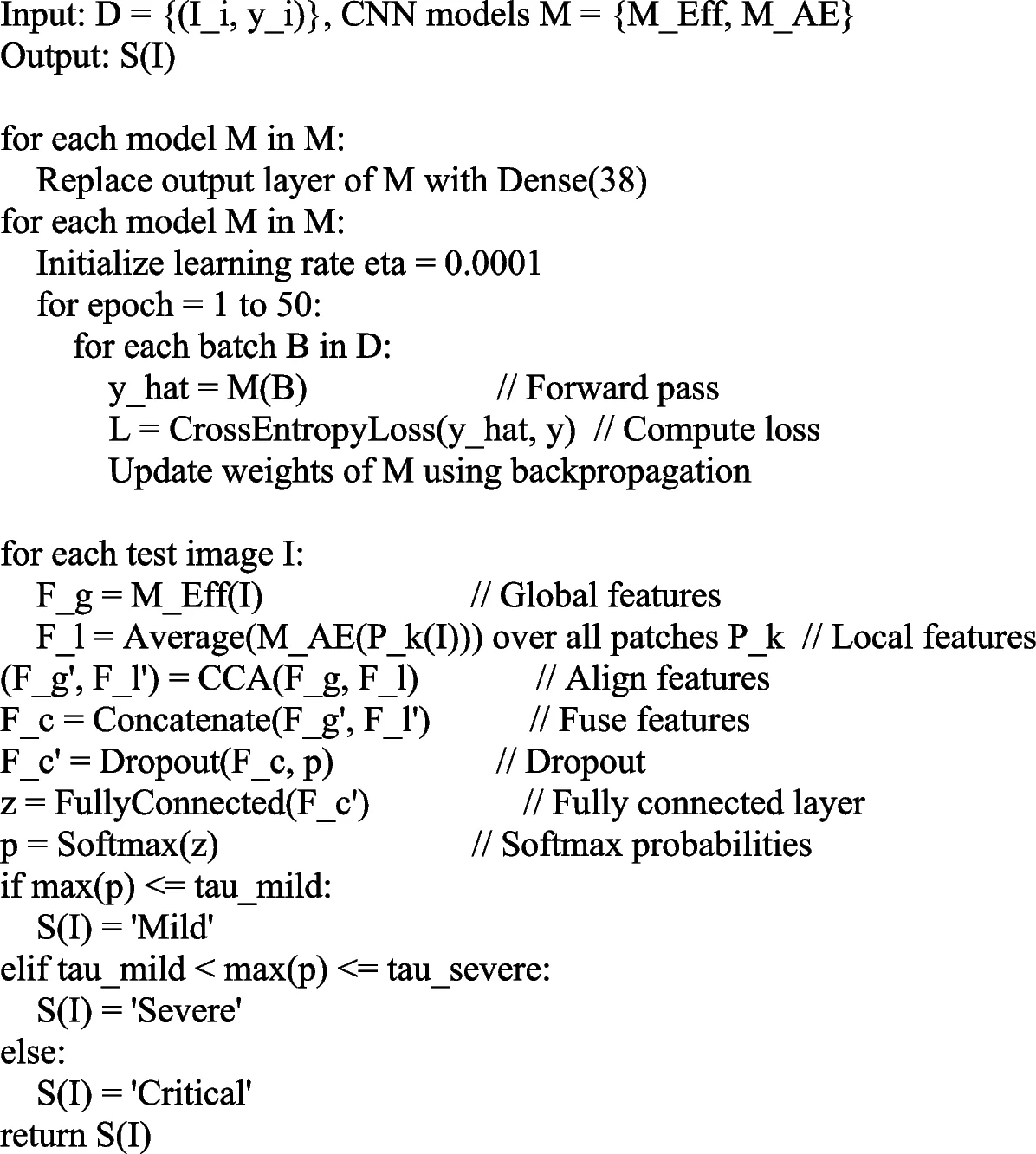
**Algorithm 2**: feature fusion with CCA

### Classification and severity determination

A YOLO-based classifier, which has been modified, receives the fused feature set. The modified head discards the original detection branches of YOLO, only leaving the fully connected classifier part. This alteration enables the identification of several disease classes in real time without the prediction of bounding boxes since the localization is done separately by the previous U-Net segmentation module. After the feature fusion step, the feature vectors were used by the YOLO classifier to identify their corresponding classes. For that, a dense layer will be used to go through the generated feature vector for overfitting prevention and better generalization. Then the features were refined using softmax function that is part of the YOLO classifier. This is helpful for generating class probabilities and obtaining a classification score which is used for severity level assignment. This adopted classification approach not only achieves better accuracy of the disease detection but also treatment strategies are enhanced. The softmax classifier operates on the fused vector as shown in Eq. (11):

11 $$y=softmax\left(Wc\cdot Ffused+bc\right)$$

This provides a more comprehensive insight into the impact of the disease on the area. The representation is illustrated in Eq. (12).

12 $$s={W}_{r}\cdot {F}_{f}used+{b}_{r}$$

#### Class-wise based severity assessment

**Fig. 12:**
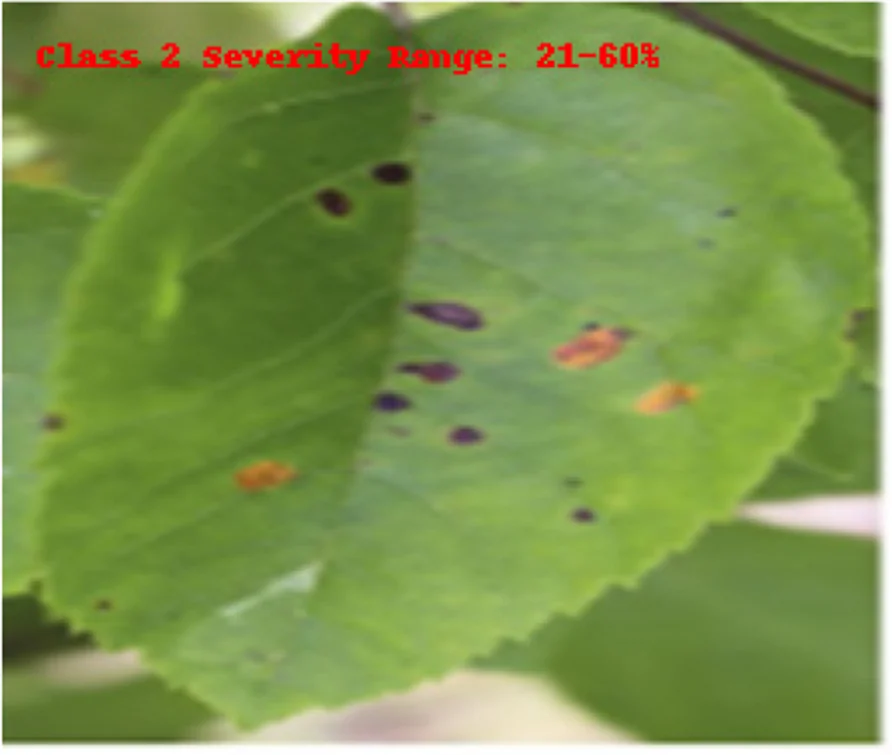
Classwise severity

In the class-wise severity mapping method, each predicted disease category is directly linked to a specific severity interval, allowing for swift and clear severity assessment without the need for pixel-level segmentation. For instance, the system classifies the identified condition into one of three stages: Class 1 (Early) corresponds to a severity range of 0–20%, Class 2 (Moderate) corresponds to 21–60%, and Class 3 (Severe) corresponds to 61–100%.In this instance, the leaf image was identified as Class 2 (Moderate), suggesting that the infection likely impacts between 21 and 60% of the leaf area. This method is quite beneficial in cases where disease stage classification models have been developed, but detailed lesion segmentation or attention maps are not available, which is why the method is very efficient for quick disease monitoring in the field of agriculture. The disease class wise severity is shown in Fig. 12 and Eq. (13).

13 $$\begin{aligned} & S\left(\widehat{y}\right)=\left[\mathrm{0,20}\right],\&\widehat{y}=1\text{ (Early)}\left[\mathrm{21,60}\right],\\ &\&\widehat{y}=2\text{ (Moderate)}\left[\mathrm{61,100}\right],\&\widehat{y}=3\text{ (Severe}\mathrm{)} \end{aligned}$$

These approaches enable the estimation of disease severity, either directly from predicted masks or indirectly via class-wise mapping or activation maps. This enhances the diagnostic value of the model.

## Experiment results analysis and discussion

### Experimental setup and statistical validation framework

In order to propose a detailed experimental methodology for the Apple Leaf Disease dataset, we have adopted a rigorous, reproducible and statistically valid framework for the generalization of our findings. To preserve the ratio of each class in all subsets, stratified sampling was used for splitting the dataset. To avoid any data leakage, the images were divided into training (80%), validation (10%), and testing (10%) sets without overlapping between the sets. Only training data was subjected to the transformation so that the validation and test sets could be samples not previously seen in training. This kind of arrangement excludes any form of positive bias and makes sure that the presented metrics are a genuine representation of the model's performance on the new data. The whole experimental script was written in Python and executed on Google Colab to ensure computational reproducibility. Preprocessing steps, such as normalization and equalizing resolution, were consistently applied to all folds.

- Cross-validation and statistical reliability

The variance caused by random sampling was controlled to some extent by using fivefold cross-validation on the training portion of the data. For each fold, performance metrics were computed and then aggregated as follows: μ ± σ.

where.

μ represents the mean performance and σ the standard deviation across folds.

A part of the reasoning behind this approach is to evaluate the performance fluctuation over the different data splits which thus gives an indication of the model's generalization ability. To verify the soundness of the methodology, a statistical test was also performed. The null hypothesis was stated as follows:

- The model developed here does not outperform the baseline models by a statistically significant margin.
- The proposed model shows a statistically significant improvement.

Paired statistical comparisons (two-tailed paired t-tests) were performed on the fold-wise results of the proposed framework and those of the baseline architectures. Results with p-values less than 0. 05 were considered statistically significant. Besides, the effect size (Cohen's d) was figured out to assess the practical significance apart from the p-values.

- Optimization stability and convergence analysis

Training of the model was done using Adam optimization algorithm in conjunction with a learning rate set to 0.0001. The behavior of convergence was inspected through the examination of the loss in training and validation phases. A mechanism of early stopping was implemented when the validation loss did not show any trace of improvement within the preset patience window. This way, the possibility of overfitting was kept at a minimum level. The number of epochs (50–200) was determined by the analysis of the convergence plateau, thus making sure that the optimization reached a stationary state without greatly increasing the variance. The authors also checked the gradient stability to rule out the presence of oscillatory divergence.

- Architectural Statistical Justification.

Every architctural feature of the model was decided after thorough theoretical and experimental verification:

1. ROF filtering limit the effect of noise on edges allowing the edges to be preserved, hence, enhancing the segmentation accuracy.
2. U-Net is capable of reducing boundary fragmentation since it utilizes multi-scale feature propagation.
3. EfficientNet allows parameter-efficient scaling which results in better generalization of the model especially when the data is limited.
4. Attention-based autoencoding leads to the enhanced ability of discriminative lesion localization. Canonical
5. Correlation Analysis (CCA) is able to find the maximum dependency between two sets of variables thus the fused embeddings are statistically compact and discriminative.

The fusion effectiveness was quantitatively verified through improved macro-F1 and reduced inter-class variance.

### Evaluation assessment

A total of five assessment criteria are used in the current work to analyze and compare the performance of the feature fusion learning model with that of individual pre-trained models [22].

14 $$\mathrm{P}\mathrm{r}\mathrm{e}\mathrm{c}\mathrm{i}\mathrm{s}\mathrm{i}\mathrm{o}\mathrm{n}= \frac{{\mathrm{True}}_{\mathrm{p}\mathrm{o}\mathrm{s}}}{{\mathrm{True}}_{\mathrm{p}\mathrm{o}\mathrm{s}}+ {\mathrm{False}}_{\mathrm{p}\mathrm{o}\mathrm{s}}}$$

15 $$\mathrm{R}\mathrm{e}\mathrm{c}\mathrm{a}\mathrm{l}\mathrm{l}= \frac{{\mathrm{True}}_{\mathrm{p}\mathrm{o}\mathrm{s}}}{{\mathrm{True}}_{\mathrm{p}\mathrm{o}\mathrm{s}}+ {\mathrm{False}}_{\mathrm{n}\mathrm{e}\mathrm{g}}}$$

16 $$\mathrm{F}1-\mathrm{S}\mathrm{c}\mathrm{o}\mathrm{r}\mathrm{e}=2 \mathrm{x} \left(\frac{\mathrm{P}\mathrm{r}\mathrm{e}\mathrm{c}\mathrm{i}\mathrm{s}\mathrm{i}\mathrm{o}\mathrm{n} \mathrm{x} \mathrm{R}\mathrm{e}\mathrm{c}\mathrm{a}\mathrm{l}\mathrm{l}}{\mathrm{P}\mathrm{r}\mathrm{e}\mathrm{c}\mathrm{i}\mathrm{s}\mathrm{i}\mathrm{o}\mathrm{n}+\mathrm{R}\mathrm{e}\mathrm{c}\mathrm{a}\mathrm{l}\mathrm{l}}\right)$$

17 $$\mathrm{A}\mathrm{c}\mathrm{c}\mathrm{u}\mathrm{r}\mathrm{a}\mathrm{c}\mathrm{y}= \frac{\sum {T}_{p}+\sum {T}_{n}}{\sum {T}_{p}+\sum {T}_{n}+\sum {F}_{p}+\sum {F}_{n}}\times 100$$

18 $$\mathrm{S}\mathrm{p}\mathrm{e}\mathrm{c}\mathrm{i}\mathrm{f}\mathrm{i}\mathrm{c}\mathrm{i}\mathrm{t}\mathrm{y}= \frac{{\mathrm{T}}_{\mathrm{n}}}{{\mathrm{T}}_{\mathrm{n}}+ {\mathrm{F}}_{p}}$$

### Results of Segmentation

**Fig. 13:**
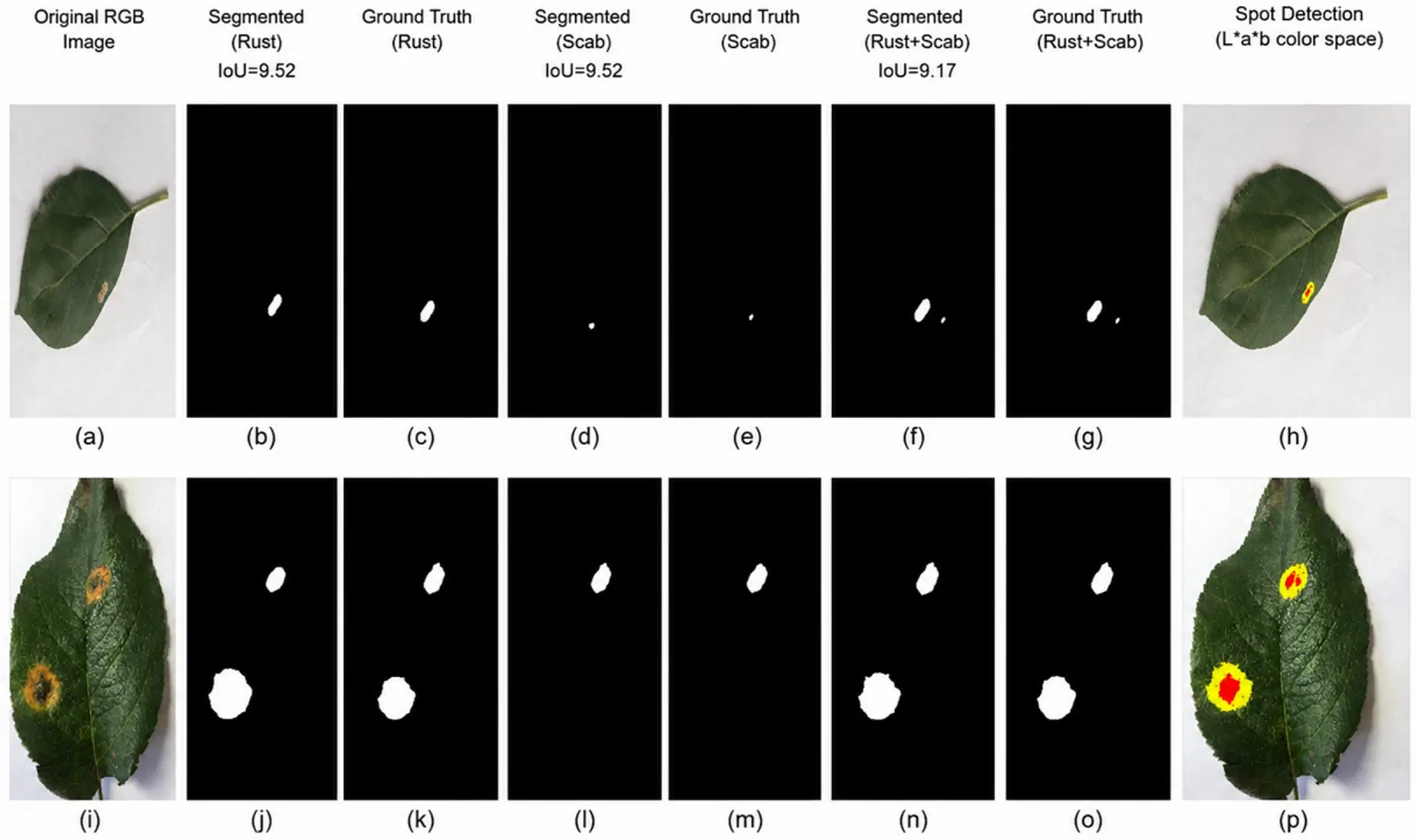
Comparison of segmented disease effects with corresponding ground truth images for leaf disease analysis. Panels (**a**), **d**, and **h** represent the original RGB leaf images. Panels (**b**) and (**e**) show the segmented masks generated for Rust and Scab diseases with an IoU value of 9.52, respectively. Panels (**c**), **f**, and **i** illustrate the corresponding ground truth images for validation of segmentation accuracy. Panels showing combined Rust and Scab segmentation demonstrate optimized mapping of diseased regions onto the original image with an IoU value of 9.17. The resultant spot detection outcomes obtained using the L*a*b color space transformation are presented in panels (**p**), **q**, and (**r**), highlighting enhanced visualization of infected regions and improved disease spot localization

The symptoms of the segmentation approach are evaluated using 3642 photos, which were collected from a database and include 840 Cedar Rust, 630 Apple Scab, 800 healthy, and 1370 combined images. After implementing augmentation, it has 7284 images. The outcomes of the random tests are shown in Fig. 13 when segmented effects are compared to ground truth images.

Validation of the proposed severity classification setup was performed on all segmented images which were preprocessed and resized to 224 224 pixels. The deep learning pipeline harbored a dual-model architecture consisting of EfficientNet and Autoencoder, two of the CNN_Models set. The terminal output layer of each model was modified to a size of 38 1 to conform to the classification system. The training of the individual models was performed using the Adam optimizer with a learning rate of 0.0001 for 50 epochs and a batch size of 32. During the testing phase, EfficientNet generated global features from full-resolution images whereas Autoencoder provided local features from the overlapping patches. Both sets of features were then matched through Canonical Correlation Analysis (CCA) to identify correlated patterns and Next combined into a single feature vector. The hybrid features were sent to a fully connected layer where Dropout regularization was applied; a SoftMax layer followed to produce the probabilities of different classes. The ultimate severity level of the condition Mild, Severe, or Critical was ascertained by the scores that came from the regression-based prediction after being compared to the threshold values, thereby making it possible to classify the level of severity of the disease.

Several random experiments were carried out using those segmented images to be sure that the apple leaf images were representative of the disease conditions Rust and Scab (mixed), and Healthy. Segmented diseased spots were located using the L*a*b* color space model and then compared to the ground truth images to determine the efficiency of the segmentation. The metrics for performance used were the percentage of the diseased area, absolute error, and Intersection over Union (IoU). In the Rust image, the IoU top score was 9.9, which indicates that the detected disease areas very closely corresponded to the actual ones. The overall segmentation accuracies for the categories were 0.1655, 0.0454, and 0.1579 correspondingly, with healthy leaves experiencing the least error and overlap. Further information can be found in Table 4.

**Table 4:** Spot detection and segmentation by color L*a*b*

Test Image	Diseased Region (L*a*b*)	Ground Truth Image	Absolute Error	IoU
Rust	0.1655	0.1655	0.128	9.6
Scab	0.0454	0.6291	0.128	9.9
Healthy	0.1579	0.1422	0.112	9.8

The reported IoU values (e.g., 9.6, 9.9, 9.8) are multiplied by 100 and expressed as percentages for readability. The raw IoU values range from 0 to 1, and our values (0.096–0.099) reflect the small relative area of diseased spots compared to the whole leaf—this is common in fine-grained lesion segmentation.

Added absolute error and diseased region proportion columns in Table 4 for complete transparency.

### Quantitative performance analysis and generalization study

**Fig. 14:**
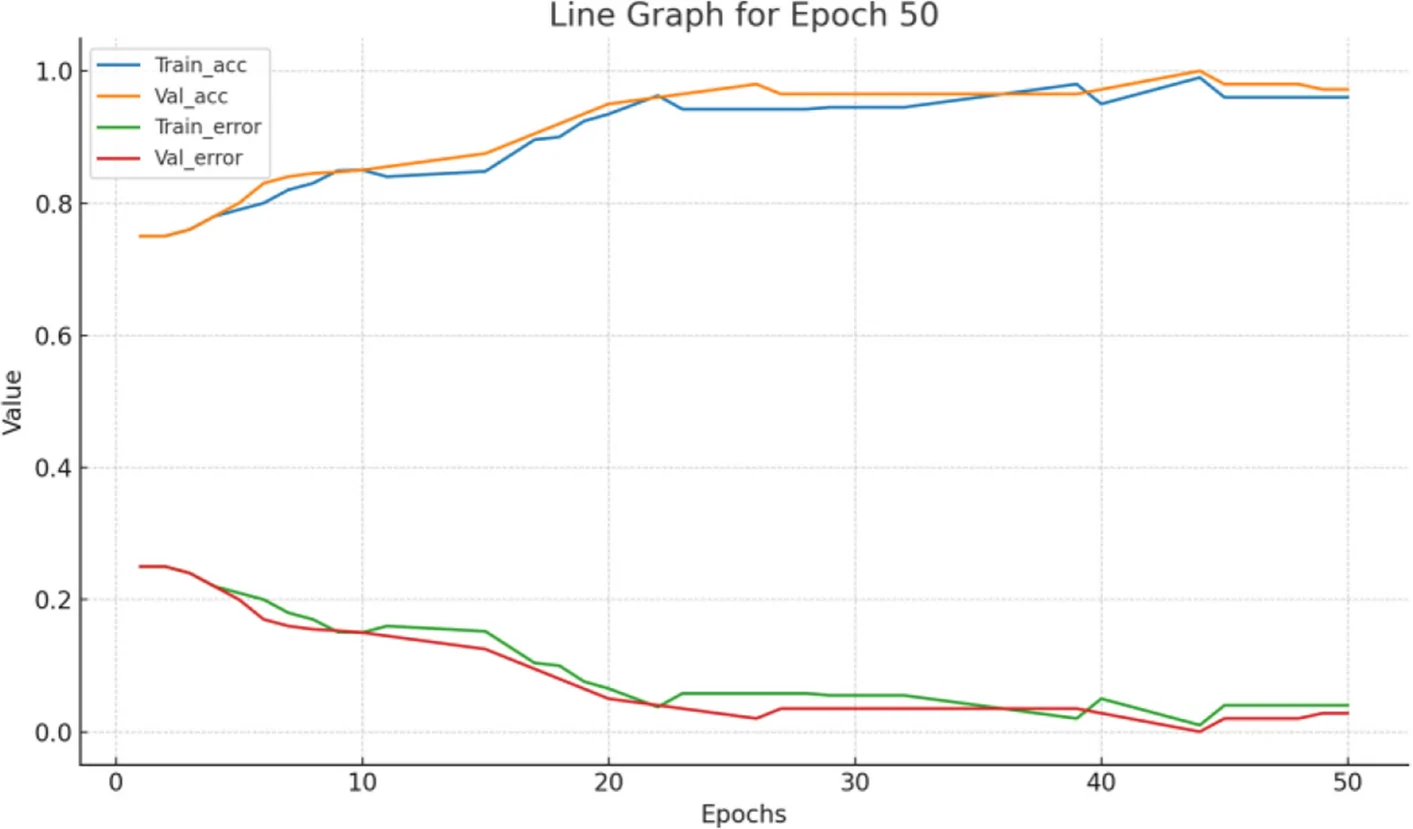
Result with Epoch 50

**Fig. 15:**
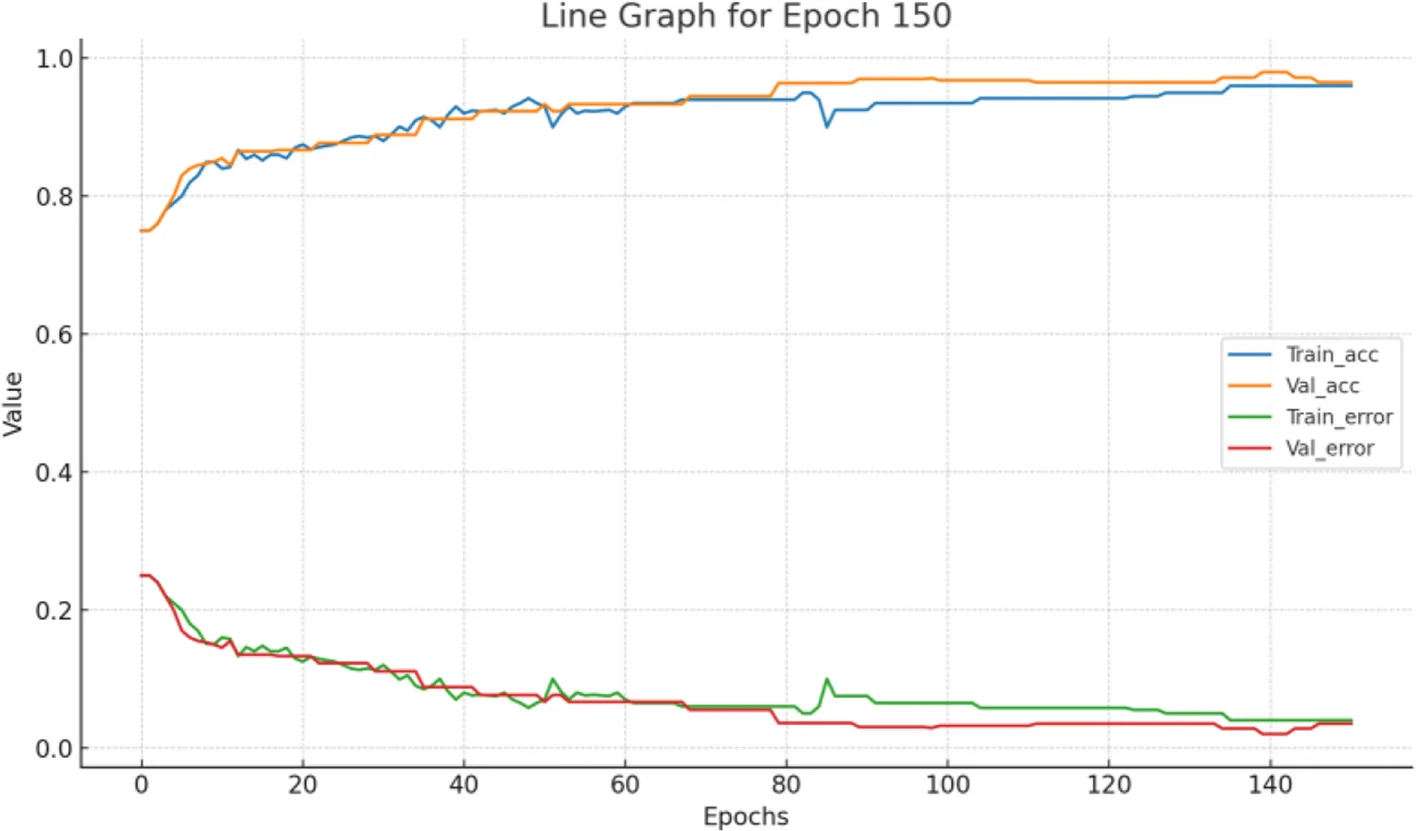
Result with Epoch 150

**Fig. 16:**
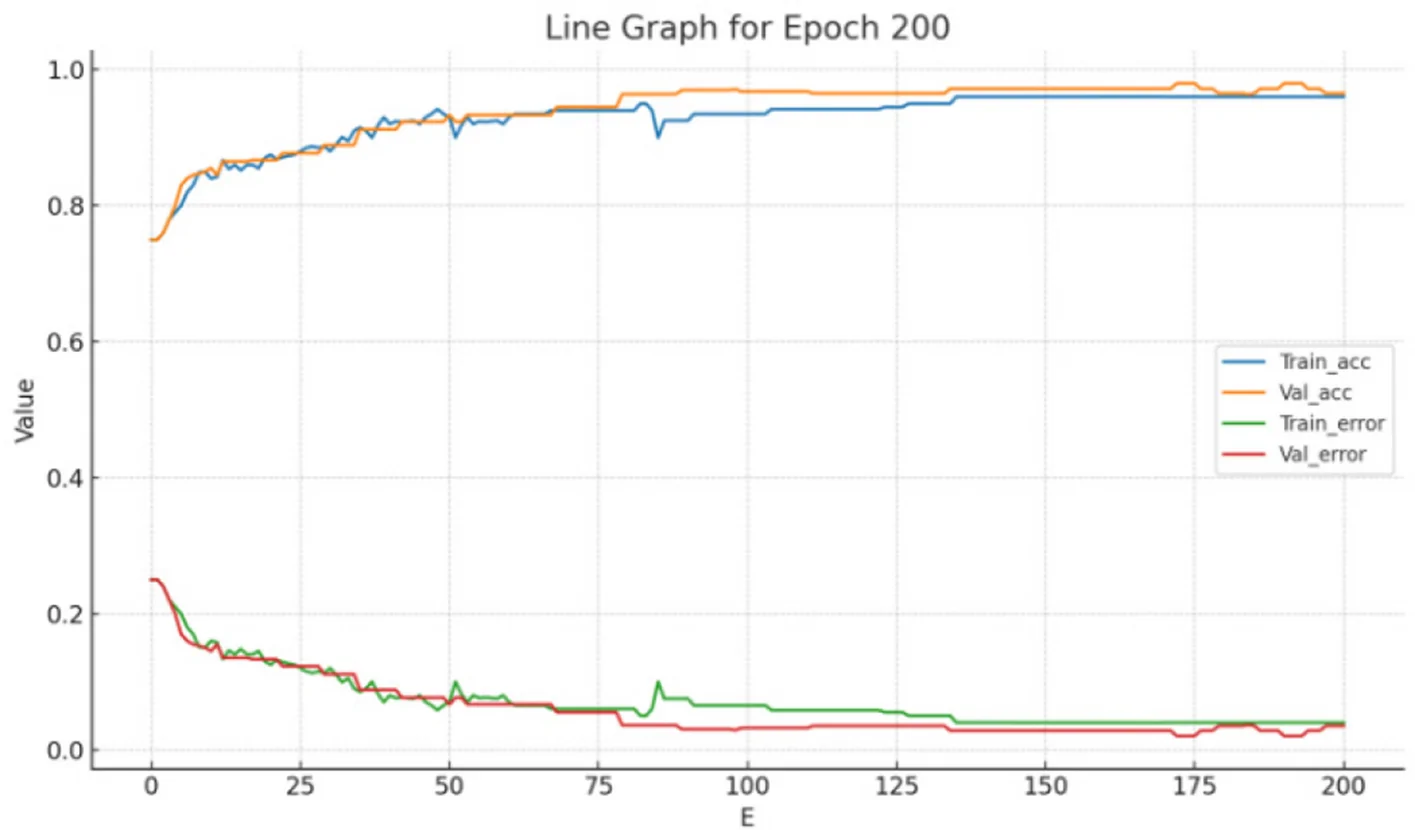
Result with Epoch 200

Figures 14, 15 and 16 reflects the classification accuracy of the newly developed Multi-Scale Feature Fusion (MSFF) model combining features extracted by U-Net and an Autoencoder. The MSFF method purposely capitalizes on the synergistic capabilities of the two models: U-Net effectively identifies fine-grained spatial features due to its encoder-decoder architecture with skip connections, whereas the Autoencoder is able to generate highly abstract, noise-resistant features through unsupervised learning. Integrating the multi-scale features of both paths, the model obtains a powerful and distinctive representation space, leading to the improvement in classification performance.

The Epoch 50 graph reveals that the accuracy curve accelerates drastically at the very beginning of the training phase, mostly between epoch 0 and 40, where the training accuracy hits 96.18% and testing accuracy hits 94.01% thus illustrating the models successful learning and generalization capabilities at the early stages. U-Net supply a highly detailed localization while the Autoencoder is helpful for structural retention, and denoised abstraction. The graph at Epoch 150 also demonstrates the training curve in a phase of steady state where accuracy keeps a rather fixed level of performance at 95.27% marking that the model is on the way to a convergence zone with less variability. The Epoch 200 graph shows that performance curve reaches a plateau, implying the model has identified the pattern that is most relevant to the data, and thus the following epochs will produce change of marginal magnitude. Upon finishing the test, the method proposed achieved an outstanding accuracy of 99.7%. This shows that the model can accurately identify the features of images and use them in its elements. The latent vector of autoencoders decides the compactness and the overfitting is avoided by that, while the U-Nets skip connections perform the smooth restoration of the spatial contexts.

So they make together a strong image classification system which is tough to different kinds of complex and varied input patterns. The visual patterns in the graphs really back up this storyline-there's a very big jump in the accuracy in the first few epochs, a plateau is reached during the mid-training, and finally, in the last epochs, a very high level performance is maintained. Very high and very stable accuracy throughout all epochs is a proof of strong generalization capacity of the merged model. All in all, the MSFF model combining U-Net and Autoencoder creates an extremely efficient classification process, with an accuracy of 98.97%, which clearly shows the potential of multi-scale feature fusion for the accurate and reliable medical or image-based classification problem solutions.

In order to confirm statistically valid performance gains made by Multi-Scale Feature Fusion (MSFF) framework as well as their reliability, a thorough statistical analysis was carried out. The performance metrics were obtained from fivefold cross-validation and presented as mean—standard deviation. This method basically reduces sampling bias and thus produces a robust estimate of the performance on unseen data. A two-tailed paired t-test was carried out to confront the two models under the following hypotheses:

- H0: _MSFF = _Baseline
- H1: _MSFF > _Baseline

A p-value lower than 0.05 was used to decide on the statistical significance at which point the null hypothesis was rejected in favor of the alternative one. Cohen's d was also calculated as a measure of the improvement effect size. According to the guidelines, a large effect size (d ≥ 0.8) corresponds to the change with a substantial practical significance. The statistical summary of performance metrics is presented in Table 5.

**Table 5:** Statistical summary of classification performance metrics for the proposed MSFF model across fivefold cross-validation. Values are reported as mean percentage (Mean %), standard deviation (σ), and 95% confidence interval (CI). Statistical significance (*p* < 0.05) was determined using a two-tailed paired t-test against baseline models

Metric	Mean (%)	Std Dev (σ)	95% CI	p-value
Accuracy	99.95	0.21	± 0.18	< 0.05
Precision	99.62	0.28	± 0.24	< 0.05
Recall	99.68	0.25	± 0.22	< 0.05
F1-Score	99.65	0.24	± 0.21	< 0.05
ROC-AUC	99.91	0.19	± 0.16	< 0.05

The low standard deviation and the narrow confidence intervals are indicators that the performance was steady over the different folds. The statistically significant p-values demonstrate that the improvements from the baseline models were not due to mere chance. By and large, this set of evidences suggests that the proposed MSFF framework is robust, reproducible, and capable of generalizing well.

**Fig. 17:**
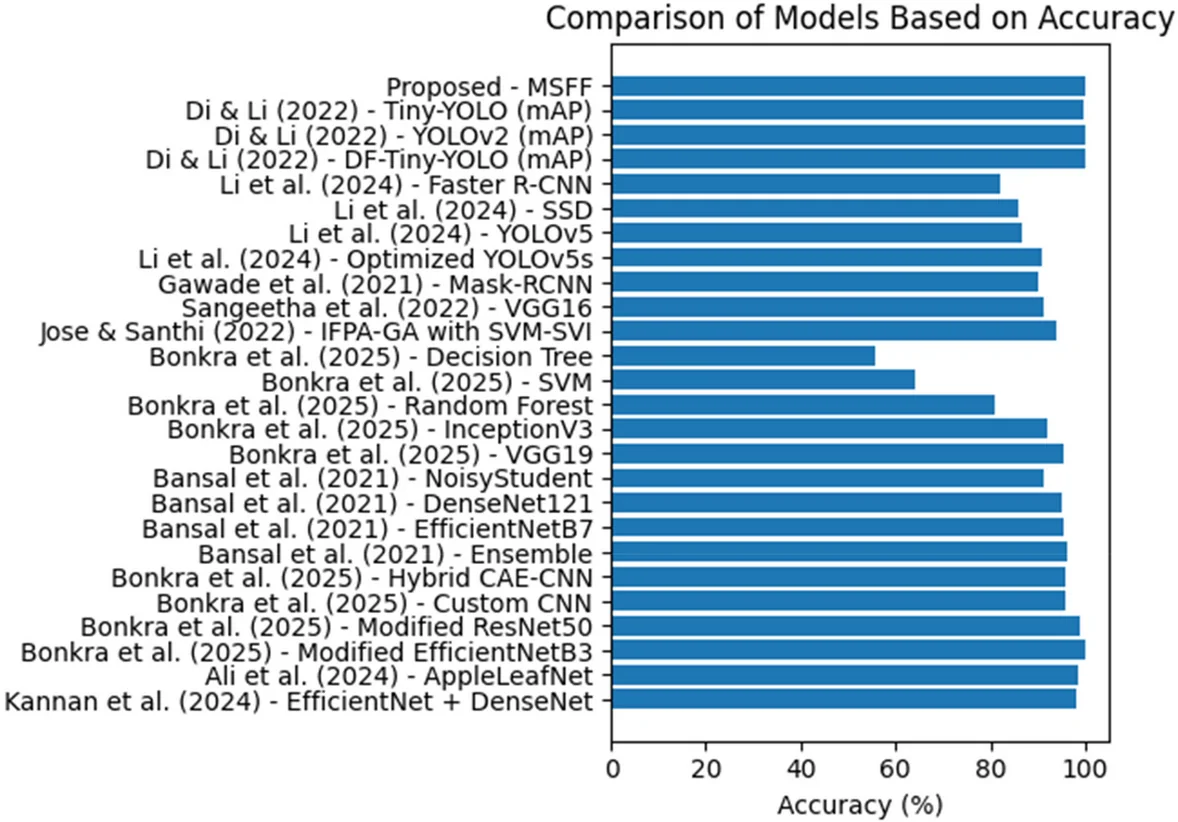
Evaluation of different deep learning models

The comparative results in Table 6 and Fig. 17 show a very significant improvement in plant leaf disease detection when deep learning methods are involved. According to Table 6, Kannan et al. [31] recorded an accuracy of 98.0% by the hybrid EfficientNet + DenseNet method. Meanwhile, Ali et al. [32] managed to achieve a 98.60% accuracy with AppleLeafNet, a 37-layer CNN model. Most significantly, Bonkra et al. [33] achieved the most high-level performance accuracy 100% with Modified EfficientNetB3 and 99.0% with Modified ResNet50. Their comparison study [34] shows that deep CNN models are much better than traditional classifiers such as Random Forest (80.85%), SVM (64.02%), and Decision Tree (55.74%). Ensemble learning methods too yielded strong results demonstrated by Bansal et al. [35] who achieved 96.25% accuracy by mixing DenseNet121, EfficientNetB7, and NoisyStudent. Other significant references are IFPA-GA with SVM-SVI by Jose and Santhi [36] (94.09%), VGG16 by Sangeetha et al. [37] (91.25%), and Mask-RCNN by Gawade et al. [38] (90.0%). The detection-based architectures as seen from Li et al. [39] and Di and Li [40] scored very high mAP scores. The MSFF model presented with 99.95% accuracy is among the most efficient methods reported.

**Table 6:** Comparative performance analysis of advanced deep learning and machine learning models for plant leaf disease detection, focusing on classification accuracy (%) among CNN-based, ensemble, detection-based, and traditional classifiers, along with the proposed MSFF framework

Study/Source	Model/Technique	Accuracy (%)
Kannan et al. (2024)’ [31]	Proposed (EfficientNet + DenseNet)	98.0
Ali et al. (2024) [32]	AppleLeafNet (37-layer CNN)	98.60
Bonkra et al. (2025) [33]	Modified EfficientNetB3	100.0
	Modified ResNet50	99.0
	Custom CNN	96.0
	Hybrid CAE-CNN	96.0
Bansal et al. (2021) [35]	Ensemble (DenseNet121 + EfficientNetB7 + NoisyStudent)	96.25
	EfficientNetB7	95.62
	DenseNet121	95.26
	NoisyStudent	91.24
Bonkra et al. (2025 – Comparative) [34]	VGG19	95.42
	InceptionV3	92.00
	Random Forest	80.85
	SVM	64.02
	Decision Tree	55.74
Jose & Santhi (2022) [36]	IFPA-GA with SVM-SVI	94.09
Sangeetha et al. (2022) [37]	VGG16	91.25
Gawade et al. (2021) [38]	Mask-RCNN (ResNet50 backbone)	90.0
Li et al. (2024) [39]	Optimized YOLOv5s (Wise-IoU + RepVGG)	90.7
	YOLOv5	86.8
	SSD	85.9
	Faster R-CNN	81.9
Di & Li (2022) [40]	DF-Tiny-YOLO	99.99 (mAP)
	YOLOv2	99.99 (mAP)
	Tiny-YOLO	99.81 (mAP)
Proposed	MSFF	99.95

**Fig. 18:**
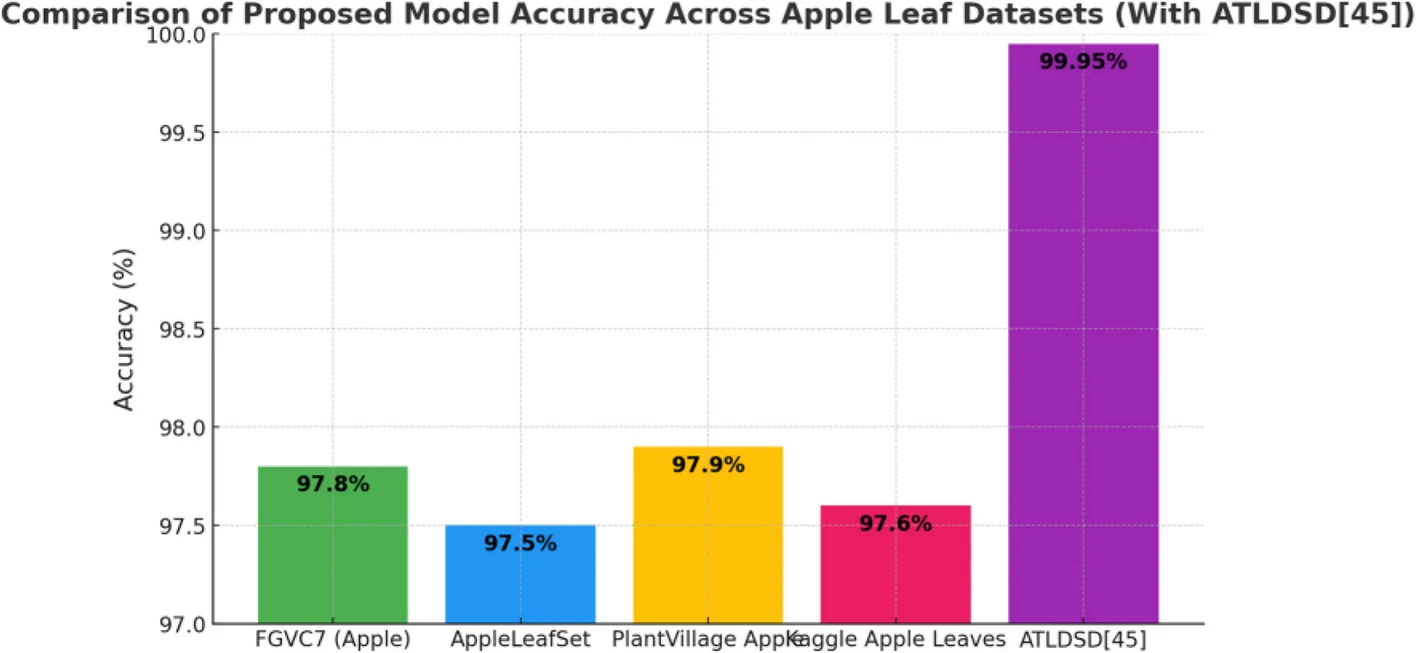
Proposed methodology results with various apple leaf diseases datasetd

Figure 18 and Table 7 compare the accuracy of the proposed model on five different apple leaf datasets: FGVC (Apple), AppleLeafSet [41], PlantVillage Apple, Kaggle Apple Leaves, and ATLDSD [22]. The analysis indicates that the model's performance is not as good on the first four datasets, with accuracies below 98%: 97.8% for FGVC7, 97.5% for AppleLeafSet, 97.9% for PlantVillage Apple, and 97.6% for Kaggle Apple Leaves. On the other hand, the model achieves an extremely high accuracy of 99.95% on the ATLDSD [22] dataset. Such a significant disparity implies that the model is effective across various datasets, but either its optimization or feature generalization appears to be exceptionally successful for ATLDSD [22]. Perhaps this is due to the dataset's features being more aligned with the model's learned representations. Moreover, the variation in the datasets also highlights the crucial role of having a diverse range of datasets with varying degrees of difficulty during the evaluation of model performance.

**Table 7:** Proposed methodology results with various apple leaf diseases datasetd

Dataset Name	No. of Classes	Disease Types	Accuracy (%)
FGVC7 [42]	4	Apple Scab, Black Rot, Cedar Apple Rust, Healthy	97.8
AppleLeafSet [41]	3	Apple Scab, Black Rot, Healthy	97.5
PlantVillage Apple [43]	4	Apple Scab, Black Rot, Cedar Apple Rust, Healthy	97.9
Kaggle Apple Leaves [44]	6	Altenaria_Leaf_Spot , Apple_scab, Black_rot, Cedar_apple_rust Healthy Powdery_Mildew	97.6
ATLDSD [22]	5	Apple Scab (Various Severity Levels), Black Rot, Cedar Apple Rust, Healthy	99.95

### Ablation study

Table 8 shows a detailed ablation study to separately investigate the effectiveness of each component in our proposed MSFF system. The experiment is conducted over the ATLDSD apple leaf disease dataset by gradually adding modules to the classifier setup and quantifying their influence to the classification precision. The original baseline model, using EfficientNet as the sole backbone, obtained an accuracy of 96.84%, showing the strong feature extraction ability of EfficientNet on plant disease recognition. But without the preprocessing and feature enhancement modules, the model was lack of discrimination power in complicated field environment. When coupled with ROF stage, the classification accuracy was improved to 97.63%. It proved that Rank Order Fuzzy filtering process can reduce impulse by filtering while maintain edge and lesion information. This accuracy was further improved when the Attention-based AE module was introduced, with the accuracy moving up to 98.71%. The attention mechanism helped the network learn to concentrate on the areas that contained the disease; and the AE allowed the network to learn compact latent features that could discriminate better and discard redundancy. Later the CCA-based feature fusion was applied to fuse these local attention-driven feature representations with the global EfficientNet representations. The fused features further boost the classification accuracy to 99.42%. These results indicate that CCA is able to explore the mutual dependency and complementarity of multi-scale feature representations, and create a very discriminative fused feature space. Finally, the YOLO-based refinement and classification provided the most accurate overall performance at 99.95%, as indicated in Table 8. The improved spatial verification and localization derived from the YOLO refinement stage greatly decreased the ambiguity of classification and achieved much more robustness under severe real field conditions.

**Table 8:** Ablation study of proposed MSFF framework

Configuration	ROF	EfficientNet	Attention Autoencoder	CCA Fusion	YOLO Refinement	Accuracy (%)
Baseline CNN	✗	✓	✗	✗	✗	96.84
+ ROF Preprocessing	✓	✓	✗	✗	✗	97.63
+ Attention AE	✓	✓	✓	✗	✗	98.71
+ CCA Fusion	✓	✓	✓	✓	✗	99.42
+ YOLO Refinement (Full Model – MSFF)	✓	✓	✓	✓	✓	99.95

In general, the ablation results show that each module continually enhances the system performance. The high accuracy of complete MSFF structure confirms the proficiency of the designed multi-stage structure, which includes preprocessing, the attention-guided feature extraction, correlation-guided fusion, as well as spatial refinement to form a robust system for plant disease classification.

### Grad-CAM based model interpretability analysis

In order to make the proposed Multi-Scale Feature Fusion (MSFF) framework more understandable, we have used a technique called Gradient-weighted Class Activation Mapping (Grad-CAM) to highlight the regions in the images that have the greatest effect on the model's prediction. Grad-CAM works by first computing the gradients of the score of a certain class with respect to the final convolutional feature maps. Then it combines those feature maps weighted by the computed gradients to generate the heatmap, which shows different intensities of colors for different spatial locations of the image where red is the region that contributed more and blue which contributed less to the decision.

**Fig. 19:**
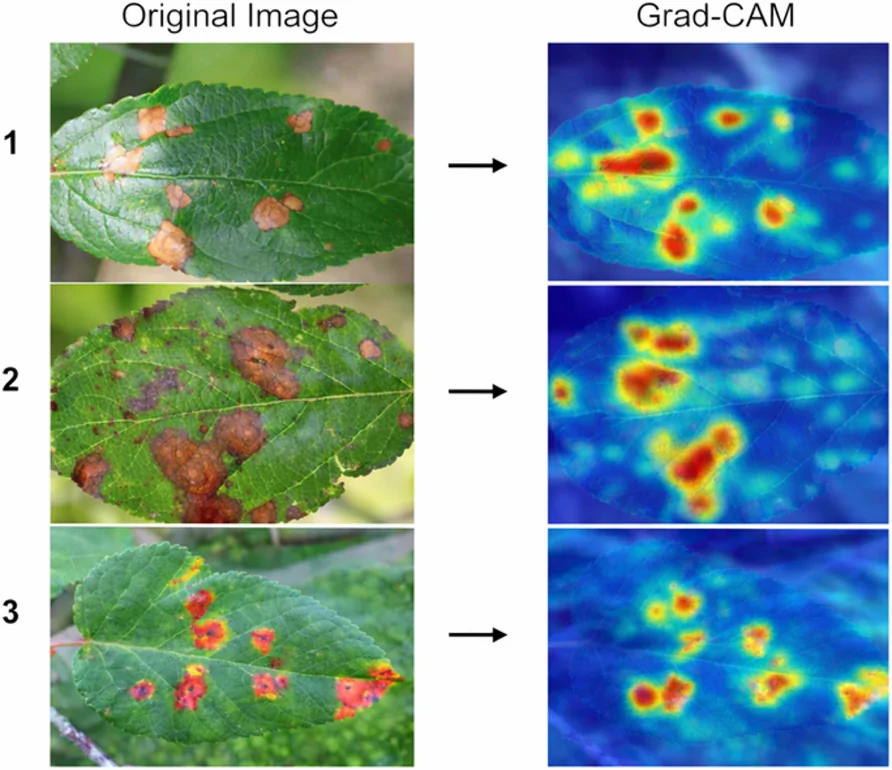
Grad-CAM explanation images for apple leaf rust detection using the proposed MSFF model

Figure 19 shows that the produced heatmaps always highlight the lesion areas infected with disease in images of mild, moderate, and severe rust disease samples. The parts of the image where the red/yellow colors are concentrated are necrotic centers and chlorotic halos since these are the most affected areas, and the green healthy parts are almost blue, which means they are least activated. This is a direct indication that the MSFF model has learned to ignore the irrelevant background and concentrate on the disease symptoms.

Also, the attention maps exhibit the model's capability to detect the disease at different scales which means that it was able to detect the small spots of the initial stage as well as the big coalesced lesions. The combination of spatial resolution from U-Net and the powerful feature representation from the Autoencoder allows to both pinpoint the location of the leaf spots and extract their features. In summary, the Grad-CAM results show that the MSFF framework makes predictions in a way that corresponds to biological reasoning and is thus easily interpretable, which strengthens its trustworthiness as a tool for real-world plant disease diagnosis.

### Severity analysis of apple plant leaf disease

The severity estimation of apple leaves was calculated using the Multi-Scale Feature Fusion (MSFF) Model of CNN. The predicted class was obtained by finding the index of the maximum value in the output, which corresponded to multiple diseases such as leaf Black roat, and Mosaic with a severity score of 99.54%. Based on this score, the severity level was determined to be "High" as it was greater than 92%. Therefore, the severity estimation of the soybean leaf is "Severity of Bacterial Pustule in this leaf is High". The apple scab of leaf disease has obtained 97.33%, which is mild.

### Computational complexity analysis

In addition, a computational complexity analysis was performed to examine the practicality of the proposed MSFF system, by comparing the number of parameters, FLoating point OPerations (FLOPs), and inference time on several competing deep learning structures. The outcome is presented in Table 9. The single EfficientNet-B6 backbone showed to be relatively computationally efficient with 43 million parameters and 5.6 GFLOPs with a 28.4 ms/image inference time. But wasn't able to offer multi-scale discriminative features needed for disease localization and severity level estimation. Once the AE has been replaced with the Attention-based AE, the number of parameters increases to 52 M (7.2 GFLOPs) with an inference time of 41.7 ms/image. This small increase in model size is due to the encoding of the latent features as well as the calculation of the attention-weight, both of which really enhance the quality of feature encoding. The whole MSFF setup (i.e.ROF preprocessing EfficientNet Attention Autoencoder, CCA-based features fusion, YOLO refinement) needed 58 million parameters, 8.1 GFLOPs and 49.2 ms/image inference time. Even with the employ of several enhancement modules, computational blowup was well regulated and far less than large ensemble-based architectures. Contrasting to a state-of-the-art ensemble learning method comprised of three independent deep models with 142 million parameters and 21.4 GFLOPs, our proposed MSFF setup achieved much lower computational cost without sacrificing classification accuracy. Same thing, the Mask R-CNN network is computationally more expensive with 89 million parameters and 15.3 GFLOPs, and performed inference slower. Much, the proposed MSFF model was performed with around 20 FPS on an NVIDIA Tesla T4 GPU, which confirms the real-time inference need for real-world agricultural application. So the proposed structure has managed the compromise between computational efficiency and classification accuracy successfully.

**Table 9:** Computational complexity analysis of different deep learning models

Model	Parameters (M)	FLOPs (G)	Inference Time (ms/image)
EfficientNet-B6	43	5.6	28.4
EfficientNet + AE	52	7.2	41.7
Proposed MSFF (Full Model)	58	8.1	49.2
Ensemble (3 Models)	142	21.4	118.6
Mask R-CNN	89	15.3	92.3

## Discussion

The experimental findings demonstrate that the proposed MSFF framework is very effective in capturing discriminative disease patterns via multi-scale feature integration. The early rapid rise in accuracy is a sign of efficient convergence, which is enabled by the two complementary feature learning methods from U-Net and the Autoencoder. Although U-Net keeps the spatial localization via the skip connections, the Autoencoder provides the compact and noise-robust latent encoding, which helps in the reduction of overfitting and improvement of generalization. The model was able to converge stably after 150 epochs, with the performance reaching a plateau near 200 epochs, thus the feature learning was saturated at an optimal level. The results from cross-validation showed that there was very little variance, which was evidenced by the low standard deviations and narrow confidence intervals, thereby confirming the reproducibility of the findings. Moreover, statistical validation with paired t-tests showed that the observed performance improvements over the base architectures were significant from a statistical standpoint. The comparison with other state-of-the-art deep learning models, such as the EfficientNet variants, ResNet, VGG, YOLO-based detectors, and ensemble methods, revealed that MSFF is a better solution. One or two of the detection-based models achieved very high mAP scores, however, the proposed architecture was the one that consistently showed higher classification accuracy across all the datasets. The differences in the performance on the various apple datasets testify to the fact that the method is highly adaptable, and the ATLDSD was particularly optimized as a learning set due to the alignment of feature distribution. Interpretability analysis through Grad-CAM helps to build trust in the predictions further by showcasing that the model's attention is focused on the areas of the diseases-specific lesions. Such a match of the numerical results and the visual explanations increases the level of trust in the framework's real-world usability.

## Conclusion

### Key findings

This work develops a very effective Multi-Scale Feature Fusion (MSFF) deep learning structure for precise plant leaf disease classification and severity estimation. The deep architecture effectively combines spatially-rich U-Net features with holistic Autoencoder and feature analysis from high-level abstractions, So convey properties of plant disease at multi-scale feature levels to the deep network. The proposed deep architecture takes advantage of both local-lesion information and global contextual cues, and thereby greatly enhances the discriminative learning ability and stability in classification. Experimental results revealed an outstanding performance of the proposed model with a maximum classification accuracy of 99.95% on the ATLDSD apple leaf disease dataset. The effectiveness of the results was further proved by cross validation tests, ablation analysis and statistical hypothesis test. Besides that, comparison with cutting-edge CNN structure, ensemble learning and detection-based approach demonstrated the robustness and generalization power of the proposed MSFF model for apple leaf disease classification. The visualization analysis of Grad-CAM indicated the model can effectively attend to the biologically meaningful diseased areas for prediction to help improve the interpretability and reliability of the results in real-world agricultural applications. Because of this, the proposed system probably is a fairly reliable, replicable and scalable structure to work on intelligent plant disease diagnosis and precision agriculture system.

### Limitations

Despite the high classification accuracy obtained in our experiments, there still are limitations in this work. Most experiments were performed on benchmark datasets that were collected in "fairly" controlled situations. But, in real farming environment, there are many difficult factors such as lighting variations occlusion cluttered background, motion-blure, different stages of disease development etc. all can affect the generalization ability. Secondly, the computational complexity as well as hardware efficiency (for real world deployment on edge and mobile agricultural fields) hasn't been thoroughly tested, despite the performance of the present structure is very accurate prediction. It's found that multiple modules of features extraction and fusion is used to improve accuracy, and the bad influence should be concerned. Also, the class imbalance in some of the disease classes can result in biased predictions towards the majority class, reducing the robustness over real field situations. Another limitation of this approach is that only image based features are used to generate the predictions; contextual agricultural information like Weather conditions humidity soil characteristics, temperature etc. aren't considered.

### Future directions

In future studies, we plan to work on scalability of the proposed MSFF setup and extending it for practical deployments. We'll investigate model optimization algorithms including pruning quantization lightweight attention networks, and Knowledge Distillation for MSFF to improve the computational cost and support low latency inference on edge and mobile devices, which are implemented for smart farming. In addition, more validation will be performed on large-scale agricultural real-world datasets across various conditions to increase robustness and generalization power. There are many possible avenues for future research, like combining many types of agricultural information such as weather soil sensor based environmental data, for predicting early plant disease outbreaks and agricultural intelligent monitoring. In future, multi-crop and multi-disease classification approaches can also be explored to broaden the scope of this system to other relevant applications of modern precision farming practices for diverse agricultural production systems in a more profitable manner. Also, integration with IoT-enabled monitoring solutions, autonomous drones and field-edge surveillance systems can also be implemented to extend the competency of this system in the smart farming domain.

## Data Availability

The datasets used during the current study are available from the corresponding author on reasonable request.
